# Motor- and cognitive-dominant functional network adaptations supporting dual-task performance in older adults

**DOI:** 10.1162/IMAG.a.1257

**Published:** 2026-05-29

**Authors:** Yan Deng, AmirHussein Abdolalizadeh, Kayson Fakhar, Tina Schmitt, Karsten Witt, Carsten Gießing, Jochem W. Rieger, Christiane M. Thiel

**Affiliations:** Biological Psychology Lab, Department of Psychology, School of Medicine and Health Sciences, Carl von Ossietzky Universität Oldenburg, Oldenburg, Germany; MRC Cognition and Brain Sciences Unit, University of Cambridge, Cambridge, United Kingdom; Institute of Computational Neuroscience, University Medical Center Eppendorf-Hamburg, Hamburg University, Hamburg, Germany; Neuroimaging Unit, School of Medicine and Health Sciences, Carl von Ossietzky Universität Oldenburg, Oldenburg, Germany.; Department of Neurology, School of Medicine and Health Sciences, Carl von Ossietzky Universität Oldenburg, Oldenburg, Germany; Research Center Neurosensory Science, Carl von Ossietzky Universität Oldenburg, Oldenburg, Germany; Applied Neurocognitive Psychology Lab, Department of Psychology, School of Medicine and Health Sciences, Carl von Ossietzky Universität Oldenburg, Oldenburg, Germany

**Keywords:** task-based functional connectivity, cognitive–motor dual-task, brain–behavior association, domain-dominant network, regularized generalized canonical correlation analysis (RGCCA)

## Abstract

Aging is associated with declines in both motor and cognitive functions, which are often examined using cognitive–motor dual-task paradigms. However, the functional brain network mechanisms supporting dual-task performance across these domains remain incompletely understood. We investigated 40 older adults (50–80 years) and 20 younger adults (20–40 years) who performed a motor single task (pedaling), a cognitive single task (Go/NoGo), and a combined cognitive–motor dual-task during functional magnetic resonance imaging (fMRI) using a custom-built MRI-compatible pedaling device. Behaviorally, older adults showed significant dual-task costs in motor performance, whereas cognitive performance was relatively preserved. At the neural level, task-based functional connectivity revealed distinct patterns of age-related network reorganization. Cognitive-dominant networks showed relatively selective connectivity increases within frontal executive and motor-planning regions, consistent with compensatory recruitment supporting preserved cognitive dual-task performance. In contrast, motor-dominant networks exhibited broader reorganization, characterized by strengthened frontoparietal control circuits but weakened cerebello-parietal and sensorimotor pathways, pointing to reduced automaticity and increased reliance on central cognitive control. Multivariate brain–behavior analyses further revealed age-related differences in latent connectivity–behavior relationships. Motor-dominant networks in older adults showed greater dispersion and stronger coupling with behavioral variability, whereas cognitive-network patterns remained largely stable and overlapping across age groups. Motor response time variability, particularly under dual-task conditions, emerged as the strongest behavioral contributor to this latent brain–behavior dimension and was associated with connectivity in frontoparietal control and motor-planning regions. Together, these findings demonstrate that aging involves an asymmetric reorganization of large-scale networks, in which motor systems become increasingly dependent on cognitive control while cognitive systems remain comparatively resilient. This network-level account identifies motor variability and cognitive–motor interdependence as sensitive markers of aging-related brain changes, with implication for understanding why cognitive–motor abilities remain stable in some individuals while declining in others.

## 1 Introduction

Aging is associated with a gradual decline in both cognitive and motor functions (Deary et al., 2009; Niederer et al., 2021; Wilson et al., 2020), which can adversely impact daily activities and independence in older adults. One informative approach for evaluating these changes is the cognitive–motor dual-task paradigm, in which individuals perform a motor task (e.g., walking) while simultaneously engaging in a cognitive activity (e.g., talking or serial subtraction). This paradigm has proven effective for detecting early signs of cognitive impairment, including mild cognitive impairment (Ali et al., 2024; Bishnoi & Hernandez, 2021; Yang et al., 2020), dementia (Collyer et al., 2022; Montero‐Odasso et al., 2018), Alzheimer’s disease (Belghali et al., 2017; Longhurst et al., 2023), and Parkinson’s disease (Kim et al., 2024; Longhurst et al., 2023). Early identification of these deficits, together with understanding their neural correlates, is critical for developing timely interventions and potentially slowing the progression of neurodegenerative conditions.

In healthy aging, findings on dual-task performance are notably inconsistent. Some studies report slower, more variable gait and greater cognitive–motor interference in older than in younger adults (Hollman et al., 2007; Lino et al., 2023). Others show relatively selective declines—for example, preserved cognitive performance but impaired motor control, such as increased gait variability (Bürki et al., 2017) or altered posture (Bohle et al., 2019). In contrast, St George et al. (2022) observed smaller walking-speed reductions in older than in younger adults, yet greater cognitive performance declines. To capture these diverse patterns, Collyer et al. (2022) proposed classifying older adults into four groups: (1) dual decline in gait and cognition, (2) gait decline only, (3) cognitive decline only, and (4) non-decliners, highlighting the behavioral heterogeneity of dual-task effects. Previous work further suggests that aging may increase functional coupling between cognitive control and motor systems in motor regulation, reflecting growing interdependence between these domains (Li & Lindenberger, 2002; Ren et al., 2013; Seidler et al., 2010). Linking such variable behavioral outcomes to their underlying neural mechanisms requires imaging approaches capable of characterizing the relative engagement and interaction of motor and cognitive brain systems during dual-task performance.

Most prior neuroimaging studies of dual tasking in older adults have relied on functional near-infrared spectroscopy (fNIRS) during walking paradigms (Holtzer et al., 2011; St George et al., 2022; Udina et al., 2021). While informative, fNIRS is restricted to superficial cortical regions, limiting insights into subcortical motor structures and deep cerebellar networks. To overcome this limitation, several MRI-compatible pedaling tasks have been developed, enabling lower-limb motor tasks that minimize head motion while allowing whole-brain measurement. These paradigms target different components of mobility, including rhythmic gait-like movement execution (Bürki et al., 2017; Promjunyakul et al., 2015; Reinhardt et al., 2020) as well as force control and continuous motor stabilization under dual-task conditions (Papegaaij et al., 2017). Together, these studies have demonstrated that pedaling-based tasks are suitable proxies for key aspects of locomotion, engaging motor, premotor, and cerebellar regions, and that dual tasking modulates both motor and prefrontal activity. However, findings remain inconsistent, with dual-task interference linked to under-, over-, or mixed activation patterns (Leone et al., 2017).

To move beyond regional activation and capture coordinated network dynamics, recent work has turned to functional connectivity approaches. A network-level perspective is well suited to dual-task research, as it enables assessment of how large-scale brain systems—particularly those supporting cognitive control and motor function—interact dynamically under varying task demands, and whether aging alters the degree of interdependence between them (Seidler et al., 2010; Vinehout et al., 2019). Yet task-based functional connectivity studies of gait-like cognitive–motor dual tasks in aging remain scarce. Most prior work has been limited to resting-state analyses, which relied on relating dual-task performance outside the scanner to changes in resting-state functional connectivity (Boyne et al., 2018; Droby et al., 2022; Yuan et al., 2015). This gap limits our understanding of how brain networks reconfigure in real time to meet concurrent cognitive and motor demands in the aging brain.

The present study addresses this gap by examining task-based functional connectivity during cognitive–motor dual-task performance in older and younger adults using an MRI-compatible pedaling paradigm. We focused on age-related neural reorganization under increasing task demands within two task-domain–dominant networks: a motor-dominant and a cognitive-dominant network defined based on task-evoked activation patterns. Importantly, the regions of interest (ROIs) used to define these networks captured relative changes in engagement from single-task to dual-task states, rather than isolating strictly separable domain-specific neural processes. To relate network-level connectivity changes to behavioral performance, we employed regularized generalized canonical correlation analysis (RGCCA), a multivariate integration approach (Rohart et al., 2017; Tenenhaus et al., 2017). This framework allowed us to identify broad multivariate associations between functional connectivity and dual-task behavior, and characterization of age-related variation in these relationships.

Our study aimed to (i) characterize age-related differences in motor-dominant and cognitive-dominant task-based functional connectivity, (ii) assess how these connectivity patterns relate to dual-task performance, and (iii) examine whether brain–behavior associations differ across age groups and task domains. Together, this framework provides new insights into how the aging brain dynamically adapts functional networks to balance resilience and vulnerability under increasing cognitive–motor demands.

## 2 Methods

### 2.1 Participants

We recruited 62 healthy, right-handed participants through the Carl von Ossietzky University of Oldenburg campus bulletin board and local advertisements. Participants were assigned to two age groups: older adults (n = 40; 19 males), aged 50–80 years (mean = 67.6, SD = 7.09), and younger adults (n = 20; 11 males), aged 20–40 years (mean = 28.0, SD = 4.87). Given the lack of established effect size estimates for task-based functional connectivity in cognitive–motor dual-task paradigms, sample size was determined based on feasibility considerations (e.g., scanner availability, funding, and recruitment demands) and by benchmarking against prior cognitive–motor dual-task neuroimaging studies (Leone et al., 2017).

Inclusion criteria were age within the specified range for the respective group and right-handedness. All participants were able to walk independently without the use of assistive devices (e.g., cane or walker) and reported good physical and mental health. Physical and mental health, as well as aspects of functional mobility, were assessed using the Short Form-12 Health Survey (SF-12; Ware et al., 1996). Demographic information, including age, gender, educational level, and medical history, was obtained through a self-report questionnaire. Exclusion criteria included any self-reported history of neurological, psychiatric, or motor disorders, use of centrally acting medication, and standard MRI contraindications (e.g., metallic implants, claustrophobia).

The study protocol was approved by the research ethics committee of the Carl von Ossietzky University of Oldenburg and conducted in accordance with the declaration of Helsinki (Drs.EK/2020/062-02). Written informed consent was obtained from all participants. Two participants from the older group were excluded due to excessive head motion that induced stripe artifacts in the fMR images.

### 2.2 Cognitive–motor dual task

#### 2.2.1 Experimental design

**Fig. 1.**
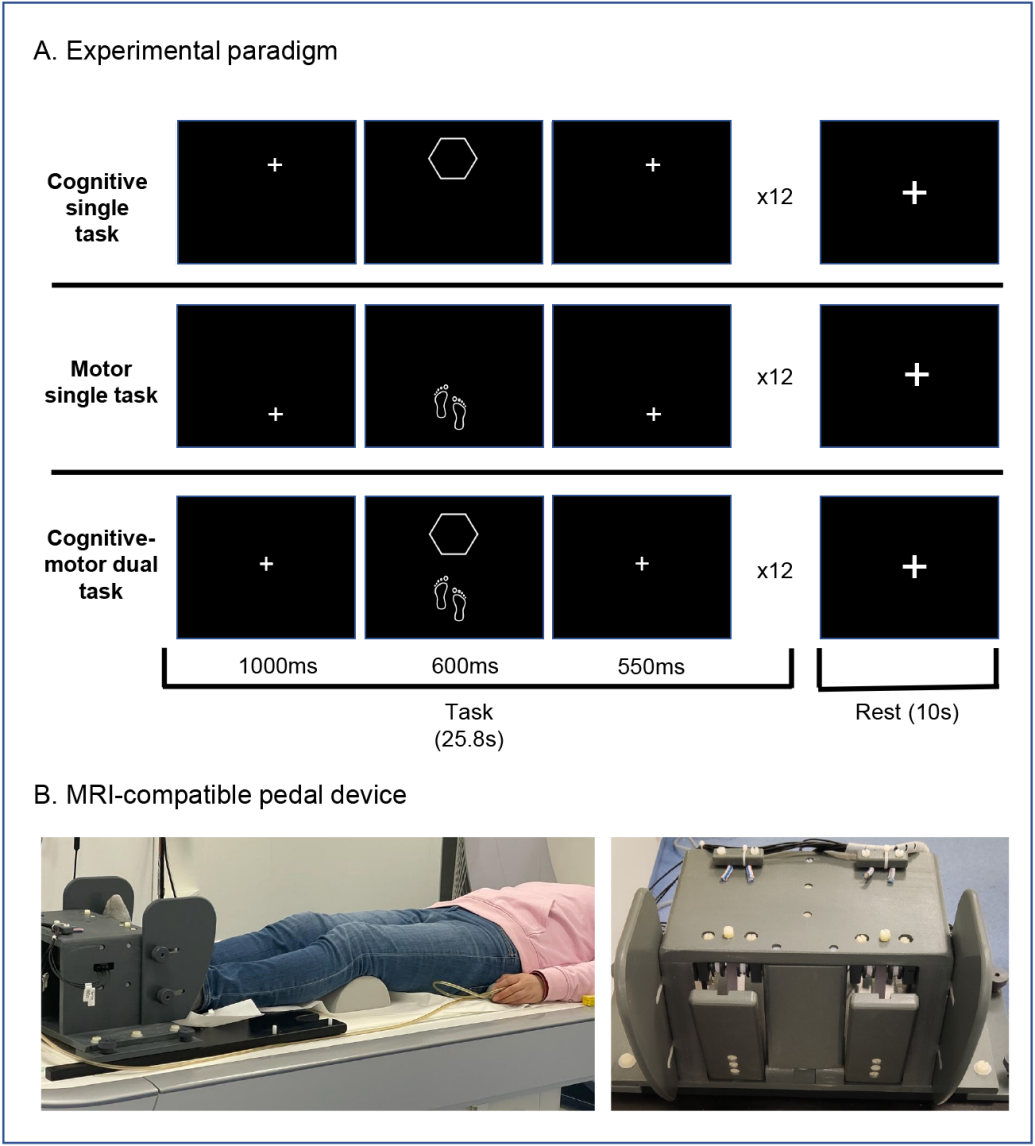
(A) Experimental paradigm: illustration of a single trial from each task condition. Upper row: cognitive single task (Go/NoGo response-inhibition paradigm requiring a button press; *“single cognitive go”*). Middle row: motor single task (lower-limb pedaling task requiring a pedal press; motor-dominant task resembling walking; *“single motor”*). Lower row: combined cognitive–motor dual task (Go trial requiring concurrent button and pedal press; *“dual go”*). Each task block consisted of 12 trials (75% Go, 25% NoGo; NoGo trials not shown), followed by a 10-s rest period. The pedaling task represents a motor-dominant task, whereas the Go/NoGo task represents a cognitive-dominant response-inhibition task. All tasks used a fixed trial duration of 2.15 s, with no temporal jitter between trials to promote rhythmic motor execution. (B) MRI-compatible pedal device: Pedals were mounted securely on a base plate and fixed within the scanner bed rails to ensure stable positioning throughout the cognitive–motor dual-task measurements. Participants were positioned comfortably on the scanner bed, and the pedal device was adjusted to their height to allow natural leg extension and optimal foot movements.

The fMRI paradigm comprised three task conditions: a cognitive single task, a motor single task, and a combined cognitive–motor dual task (Fig. 1A). The order of the two single-task runs was counterbalanced across participants; the dual-task run was always presented last. Each task began with a 10-s instruction period, followed by eight cycles of alternating task and rest periods. Task blocks lasted 25.8 s and were followed by 10-s rest periods, resulting in a total duration of 296.4 s (≈4.94 min) per task. All task conditions were implemented with fixed and predictable inter-trial intervals. The motor task was implemented at a fixed cycle time of 4.33 s per cycle (corresponding to 2.15 s per half-step), which was identical across all task conditions and participants. This cadence was not intended to reflect natural overground gait timing, which is typically faster (Hollman et al., 2011), but was selected to accommodate the constraints of fMRI acquisition. In particular, the slower, externally imposed rhythm facilitated stable head positioning during bilateral lower-limb movements and reduced motion-related artifacts. In addition, matching the temporal structure of the motor task to the externally paced cognitive task ensured comparable timing across domains. The selected cadence, therefore, represents a compromise between preserving gait-like rhythmic coordination and maintaining data quality under scanner constraints. This design ensured consistent temporal structure across tasks, facilitated stable head positioning during bilateral lower-limb movements, supported automated motor execution, and maintained inhibitory control demand in the Go/NoGo task.

Participants completed two practice sessions: (i) a computer-based session outside the scanner to ensure task comprehension and (ii) an in-scanner familiarization session to practice performing the tasks in the supine position under MRI conditions. Both practice sessions included two blocks of each task and lasted about 4.5 min per session.

#### 2.2.2 MRI-compatible pedal device

Custom-made, MR-compatible foot pedals (Fig. 1B) were used to elicit alternating dorsiflexion and plantarflexion movements, simulating gait-related activity in a supine position. The pedals, constructed from polyvinyl chloride (PVC), were securely mounted on a base plate and fixed within the scanner bed rails to ensure stable positioning throughout the measurement period (Fig. 1B). Pedal movements generated signals that were transmitted via optical fibers to an interface unit outside the scanner room, where they were converted into electrical signals and transferred via USB connection to a PC. The output included a numerical string representing the real-time position of each pedal along with a timestamp, recorded with a temporal resolution of 0.1 ms. This setup enabled precise monitoring of foot movements, allowing accurate assessment of motor performance during the experiment.

#### 2.2.3 Cognitive single task

The cognitive single task employed a Go/NoGo inhibitory control paradigm, adapted from the Human Connectome Project in Aging (HCP-A; Bookheimer et al., 2019). Participants were instructed to press a button with their right index finger as quickly as possible in response to white-outlined geometric shapes presented on a black background and to withhold responses to two specific shapes. Six shapes—plaques, trapezoids, pentagons, hexagons, octagons, and parallelograms—served as Go stimuli (*“*single cognitive Go”), while squares and circles served as NoGo stimuli (“single cognitive NoGo”). Each stimulus was presented for 600 ms in the upper half of the screen, with stimuli presented in randomized order. Each task block consisted of 12 trials and was followed by a 10-s rest period, during which a larger fixation cross was displayed. Each trial followed a fixed inter-trial interval identical to the motor and dual-task conditions, with a total trial duration of 2.15 s, and no temporal jitter was introduced between trials. The task was repeated eight times, resulting in a total task duration of 296.4 s (≈4.94 min). In total, the task comprised 96 trials, with 75% Go trials and 25% NoGo trials.

#### 2.2.4 Motor single task

In the motor single task, a foot symbol was presented in the lower half of the screen at a fixed rhythm (2.15 s per step). Participants were instructed that they could begin pedaling with either the left or the right foot and to alternate between feet in response to each symbol across trials (“single motor”). This instruction was intended to promote natural, automatic rhythmic lower-limb responses while minimizing trial-to-trial working memory demands. Pedal signals were recorded during stimulus presentation and for an additional 550 ms, capturing the full movement cycle from pedal press to release. Each stepping cycle lasted 2.15 s, comprising a 1-s fixation period (interstimulus interval), 600 ms of stimulus presentation, and 550 ms of additional pedal signal recording. Each block consisted of 12 trials, with a fixed inter-trial interval identical to that used in the cognitive and dual-task conditions. No temporal jitter was introduced between trials to encourage automated motor execution. As in the cognitive task, each motor task block was repeated eight times and followed by a 10-s rest period, during which a larger fixation cross was presented. In total, the motor single task comprised 96 trials and lasted 296.4 s (≈4.94 min).

#### 2.2.5 Cognitive–motor dual task

In the cognitive–motor dual task, the geometrical shape and the foot symbol were presented simultaneously, with shapes appearing in the upper part of the screen and the foot symbol in the lower part. Participants were instructed to respond to Go trials by pressing a button with their index finger while simultaneously stepping with one foot, either the left or the right, alternating sides across trials (“Dual Go”). As in the motor single task, participants could begin pedaling with either the left or the right foot. In NoGo trials, participants were instructed to withhold the button press while continuing the stepping movement (“Dual NoGo”). Each dual-task trial lasted 2.15 s comprising a 1-s fixation period (interstimulus interval), 600 ms stimulus presentation, and 550 ms of additional pedal signal collection. Each block included 12 trials and was followed by a 10-s rest period, during which a larger fixation cross was presented. The task was repeated across eight blocks, resulting in a total task duration of 296.4 s (≈4.94 min).

All three tasks were presented, and behavioral responses were recorded using Presentation software (version 22.1; Neurobehavioral System, Inc., Berkeley, CA).

### 2.3 Test of Attentional Performance (TAP-M) battery

After completion of the dual-task paradigm, participants performed the Test of Attentional Performance (TAP-M, version 1.3.2; Zimmermann & Fimm, 2002) to provide a general assessment of attentional and executive functions. Four subtests were administered: (1) The distractibility task assessed the ability to maintain central focus while ignoring unpredictable peripheral stimuli. The outcome measure was the mean reaction time (RT) to central targets in the presence of distractors. (2) The executive control task probed selective attention, inhibition, and cognitive flexibility through responses to specific color–letter or color–number combinations. Outcome was mean RT for correct trials. (3) The divided attention task required simultaneous monitoring of visual and auditory streams. Outcome measures included mean RTs for correct detections in each modality. (4) The Go/NoGo task assessed response inhibition, with mean RT for correct “Go” responses as the outcome measure.

### 2.4 Behavioral data analysis

All behavioral data were processed using MATLAB (version 2022b). Behavioral outcome measures included mean RT, RT variability (quantified as the standard deviation, SD, of reaction times), dual-task cost, and error rate. Cognitive performance was assessed by computing mean RT and SD across all correct Go trials, separately for the single-task and dual-task conditions. Cognitive RT was defined as the time between stimulus onset (geometric shape) and the corresponding button press. False responses to NoGo stimuli were counted as errors in cognitive performance and were calculated as the number of incorrect responses divided by the total number of NoGo trials, reported as error rate in percentage. Motor performance was gauged using mean RT and SD, calculated across all pedal presses for each participant under both single-task and dual-task conditions. Missed pedal presses were counted as motor errors and were calculated as the number of missed responses relative to the total number of expected pedal presses, reported as error rate in percentage. Motor RT was defined as the time between stimulus onset (foot symbol) and the onset of the pedal displacement. Dual-task costs for both cognitive and motor performance (based on mean RT and SD) were computed as:

DTC=[(Dual-task−Single-task)/Single-task]×100,

the relative change between dual-task and single-task performance, expressed as a percentage of single-task performance. Due to technical issues, cognitive Go/NoGo data were missing for five participants and motor pedaling data for one participant. These participants were excluded only from analyses involving the corresponding behavioral measures. For the brain–behavior RGCCA, missing values were imputed using the group mean substitution to retain participants in the multivariate analysis.

Statistical analyses focused on mean RTs and RT variability (SD) and were conducted using three-way mixed-design analyses of variance (ANOVAs) to assess the effects of age (younger vs. older adults; between-subjects), task condition (single vs. dual; within-subjects), and task domain (motor vs. cognitive; within-subjects), as well as all interaction effects. Given the unequal group sizes (40 older adults vs. 20 younger adults), Type III sum of squares were employed, as they provide unbiased estimates in the unbalanced design. Statistical significance was assessed using F-tests (α = 0.05). Effect sizes are reported as generalized eta squared. All statistical analyses were performed in R (version 4.4.3) using the *afex* package.

To illustrate age-group differences across all behavioral measures, we conducted independent-samples *t*-tests comparing younger and older adults within each task condition. To account for multiple comparisons, we applied a Bonferroni correction to adjust the significance threshold for multiple testing (16 comparisons), resulting in an adjusted significance threshold of *p* < 0.0031 (0.05 /16). As an additional post hoc analysis, pedaling cycle time (stride time) and its variability were computed to provide a gait-related measure comparable with prior pedaling studies (e.g., Bürki et al., 2017). Cycle time was calculated separately for the right and left feet as the interval between consecutive pedal events, excluding intervals exceeding 8 s to remove scheduled pauses. Mean and variability (standard deviation) were computed for each foot and then averaged to yield overall cycle time and variability for each participant. These measures were analyzed analogously to the reaction time metrics and are reported in the Supplementary Table S2.

### 2.5 fMRI data acquisition

**Fig. 2.**
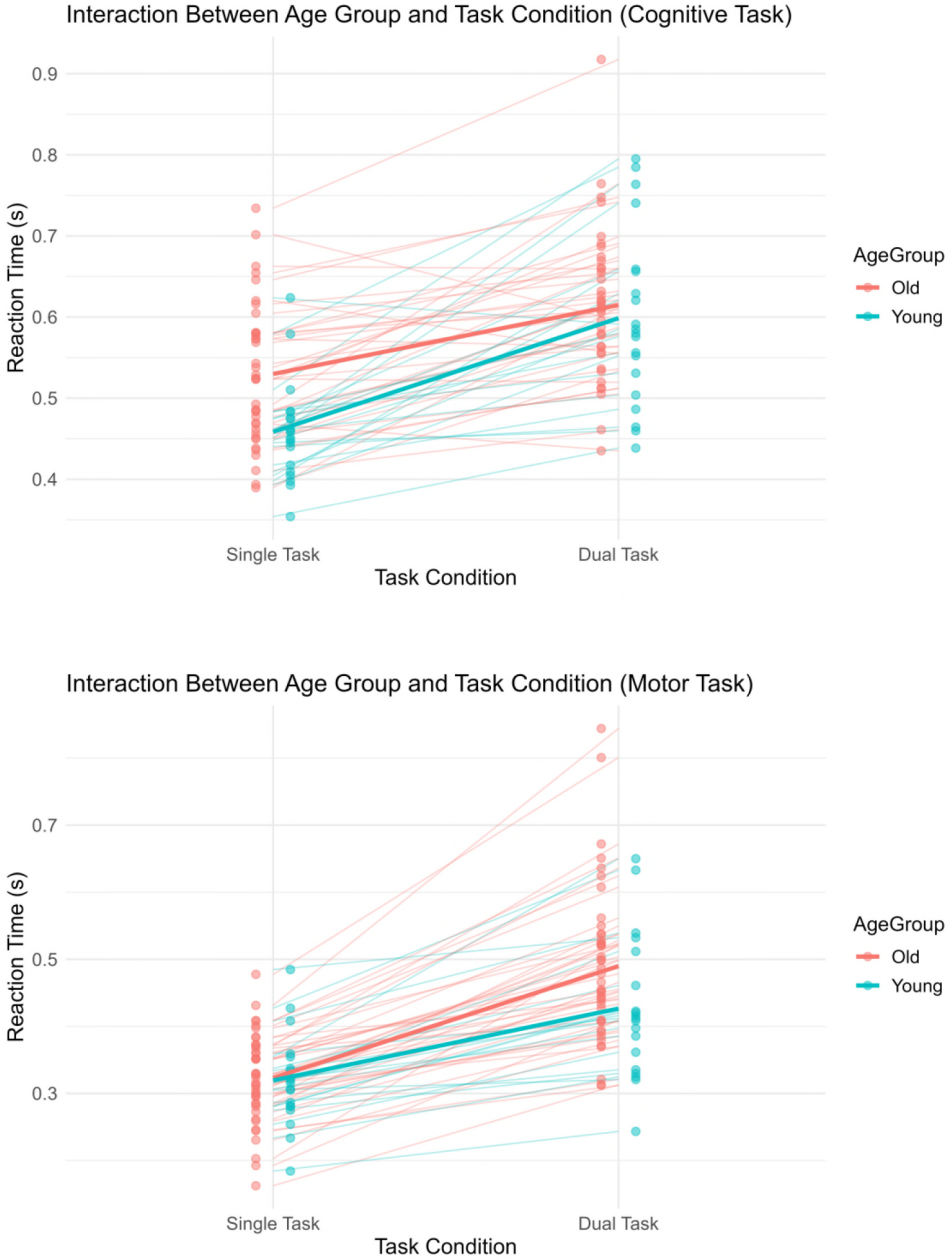
Reaction times (RTs) as a function of age (older vs. younger) and task condition (single vs. dual) for the cognitive (upper panel) and motor task (lower panel). In the cognitive task, older adults (red) responded significantly slower than younger adults (blue) in single-task conditions, but this age difference was not observed under dual-task conditions. In contrast, in the motor task, performance was comparable between groups in the single-task condition, but older adults showed significantly longer RTs than younger adults during dual-task performance. Dots and thin lines represent individual participants, and thicker lines indicate group means.

MRI data were acquired using a 3T Siemens MAGNETOM Prisma scanner (Siemens Healthcare, Erlangen, Germany) equipped with a 64-channel head coil. Participants were positioned comfortably on the scanner bed, and the pedal device was adjusted according to individual height to allow natural leg extension and effective foot movements (Fig. 2B). To minimize head movement during pedaling, participants were instructed to keep their heads as still as possible. Soft foam pads were used to stabilize the head, and a strip of fixing plaster (Leukosilk) was placed on the participant’s forehead and the borders of the head coil as a tactile aid, to facilitate self-monitoring of unintended head movements. The fMRI session began with a short training phase, during which participants practiced both single and dual tasks, which lasted approximately 4.5 min. After training, task performance was evaluated with regard to alternating pedaling and head motion, and participants received brief feedback before proceeding to the main task, which lasted approximately 15 min. Following the task and subsequent structural MRI acquisition, a resting-state fMRI scan was performed at the end of the scanning session, during which participants were instructed to keep their eyes open and fixate on a white circle presented on a black background at the center of the screen. They were asked to remain still, stay awake, and allow their thoughts to flow naturally without engaging in any specific or structured mental task. These instructions were intended to standardize visual input and minimize task-related cognitive engagement during resting-state acquisition. Resting-state data were collected for exploratory analyses and are not included in the analyses presented here.

Task-based functional images were acquired using a T2*-weighted gradient echo planar imaging (EPI) sequence with blood oxygenation level-dependent (BOLD) contrast, using a multiband sequence developed by the Center for Magnetic Resonance Research (CMRR), University of Minnesota (Feinberg et al., 2010; Moeller et al., 2010; Xu et al., 2013). Acquisition parameters were as follows: repetition time (TR) = 850 ms, echo time (TE) = 30 ms, flip angle = 62°, distance factor = 0%, slice thickness = 2.5 mm, field of view = 192 × 192 mm, matrix size = 76 × 76, voxel size = 2.5 × 2.5 × 2.5 mm³, 48 slices, multiband factor = 4, acquisition time = 15:26 min, and 1090 volumes. After completing the task, a high-resolution three-dimensional T1-weighted structural image was acquired for each participant using the following parameters: TR = 2000 ms, TE = 2.07 ms, flip angle = 9°, voxel size = 0.75 × 0.75 × 0.75 mm³, GRAPPA acceleration factor = 2, field of view = 240 × 240 mm, 224 sagittal slices, acquisition time = 6:16 min. Finally, a resting-state fMRI scan was acquired using the same multiband EPI sequence with BOLD contrast (TR = 850 ms, TE = 30 ms, flip angle = 62°, slice thickness = 2.5 mm, field of view = 192 x 192 mm, matrix size = 76 x 76, voxel size = 2.5 x 2.5 x 2.5 mm³, 48 slices, multiband factor = 4), yielding 560 volumes.

### 2.6 fMRI data analysis

fMRI data analysis consisted of three steps. First, a task-based voxel-wise general linear model (GLM) analysis (“brain activation analysis”) was performed to identify brain regions associated with cognitive-dominant and motor-dominant dual-task demands, using the contrasts Dual Go – Single Motor and Dual Go – Single Cognitive, respectively (Fig. 4). These functionally defined regions of interest (ROIs) then served as the basis for subsequent task-based functional connectivity analyses. Note that the ROIs aimed to identify networks that capture relative changes in motor- and cognitive-dominant engagement from single-task to dual-task states, rather than isolating strictly domain-specific neural processes, reflecting context-dependent network configurations. In a second step, ROI-to-ROI functional connectivity analyses were conducted within these task-dominant networks to examine differences in task-based functional connectivity between younger and older adults (Figs. 5 and 6). In the third step, we examined how individual differences in connectivity related to dual-task behavioral performance using regularized generalized canonical correlation analysis (RGCCA), referred to as “multivariate brain–behavior association analysis” (Figs. 7 and 8).

#### 2.6.1 Task-based GLM analysis and ROI selection

Functional and anatomical MRI data were preprocessed using *fMRIPrep* (Esteban et al., 2019) (version 24.1.1; RRID:SCR_016216), a fully reproducible and standardized preprocessing pipeline designed for task-based and resting-state fMRI analyses (https://fmriprep.org/en/stable/). The preprocessing workflow included fieldmap-based susceptibility distortion correction, head motion correction (realignment), coregistration of functional and anatomical images, brain extraction, tissue segmentation, and spatial normalization to MNI152NLin2009cAsym space. Full details of the preprocessing procedures are provided in the Supplementary Material A.

Following preprocessing, first-level and second-level task-based statistical analyses were conducted in SPM12 (Wellcome Centre for Human Neuroimaging, UCL, London, UK; https://www.fil.ion.ucl.ac.uk/spm/). For these analyses, functional images were spatially smoothed with an 8 mm full-width at half-maximum (FWHM) Gaussian kernel to enhance the signal-to-noise ratio for task-based analyses. Task regressors were then specified and estimated within the general linear model (GLM) framework in SPM12.

At the first-level analysis, five task regressors were specified to model trial-related BOLD responses across the cognitive, motor, and dual-task conditions. These included: (i) single cognitive Go (Go trials in the single-task cognitive condition); (ii) single cognitive NoGo (NoGo trials in the single-task cognitive condition); (iii) single motor (pedaling trials in the single-task motor condition); (iv) Dual Go (Go trials with concurrent pedaling in the dual-task condition); (v) Dual NoGo (NoGo trials with concurrent pedaling in the dual-task condition).

Each trial was modeled as a zero-duration (0 s) event time locked to the onset of the visual stimulus and convolved with the canonical hemodynamic response function (HRF) to estimate condition-specific BOLD responses. Statistical analysis was performed within the general linear model framework (Friston et al., 1994). Six realignment-derived head motion parameters (three translations, three rotations) were included as nuisance regressors to account for residual motion artifacts. To reduce physiological and scanner-related noise, a high-pass filter with a 1/128 Hz cutoff was applied, and a first-order autoregressive (AR(1)) model was applied to correct for temporal autocorrelations (Friston et al., 1995).

To define task-relevant brain ROIs for subsequent functional connectivity analyses, we computed two targeted contrasts at the first level. The dual-task cognitive-dominant contrast (Dual Go – Single Motor) captures cognitive-related engagement as expressed within the dual-task context, including contributions from dual-task control processes and context-dependent modulation relative to single-task performance. Conversely, the dual-task motor-dominant contrast (Dual Go – Single Cognitive) captures motor task-related engagement as expressed within the dual-task context, likewise incorporating dual-task control processes as well as modulation of cognitive components to their single-task baseline (see Fig. 4). These contrasts were used for ROI definition to characterize task-relevant brain regions associated with the cognitive- and motor-dominant engagement in the context of dual tasking rather than isolating pure motor or cognitive processes. Whole-brain activation maps for all single- and dual-task contrasts are reported in Supplementary Figure S1. In addition to these primary contrasts, an exploratory contrast isolating dual-task–specific activity (Dual Task − [Single Motor + Single Cognitive], DT − (SM + SC)) was computed and compared with the motor-dominant contrast (Dual Go – Single Cognitive) and Single Motor (SM) contrast (Supplementary Fig. S4).

Contrast images were entered into a random-effects general linear model (GLM) at the second level to assess task-related activation patterns across all participants (younger and older adults combined). Statistical parametric t-maps were generated, and multiple comparisons were controlled using family-wise error (FWE) correction across the whole brain. A significance threshold of *p* < 0.05 (FWE corrected at the voxel level), with a minimum cluster extent of 20 contiguous voxels, was applied to identify regions with statistically significant activation. Significant clusters for the two targeted contrasts were then extracted and used as ROIs for subsequent task-based functional connectivity analyses.

To improve anatomical specificity, each statistically defined cluster was further constrained using the Harvard-Oxford Cortical and Subcortical Structural Atlas: only voxels falling within atlas-defined anatomical boundaries were retained. Thus, clusters identified from statistical contrasts were refined by limiting them to voxels strictly within well-defined anatomical regions, ensuring that the resulting ROIs correspond to well-defined brain structures. Subclusters smaller than 20 voxels after atlas masking were excluded. This procedure yielded 47 ROIs from the cognitive-dominant contrast and 70 ROIs from the motor-dominant contrast. These ROIs were subsequently entered into ROI-to-ROI functional connectivity analyses using the CONN toolbox (Nieto-Castanon, 2020; Whitfield-Gabrieli & Nieto-Castanon, 2012). A complete list of ROIs, including anatomical labels and voxel counts, is provided in Supplementary Table S1.

#### 2.6.2 Functional connectivity analysis

##### 2.6.2.1 Preprocessing

Functional connectivity analyses were conducted using the CONN toolbox (version 22.v2407; RRID:SCR_009550; Nieto-Castanon, 2020; Whitfield-Gabrieli & Nieto-Castanon, 2012), in combination with SPM12 (release 12.7771; RRID:SCR_007037) within MATLAB 2023a, performed on a high-performance computing (HPC) cluster. The default CONN preprocessing pipeline was applied, including functional realignment and unwarping (including correction for susceptibility distortion interactions), slice-timing correction (reference to middle slice), outlier detection, tissue segmentation and normalization to standard space, and spatial smoothing. Tissue segmentation was performed directly on each participant’s T1-weighted structural image using the unified segmentation approach implemented in SPM12, which simultaneously segments the image into gray matter, white matter, and cerebrospinal fluid (CSF) compartments while computing nonlinear spatial normalization parameters. These parameters were then applied to normalize both structural and functional images to MNI152 standard space (Montreal Neurological Institute) at 2-mm isotropic resolution. Motion outliers were identified using the Artefact Detection Tools (ART) with default thresholds: framewise displacement exceeding 0.9 mm and global signal change exceeding 5 standard deviations. Spatial smoothing was applied using a Gaussian kernel with 8 mm full-width at half-maximum (FWHM).

##### 2.6.2.2 Denoising

Functional data were denoised using the standard denoising pipeline implemented in the CONN toolbox, which involves three key sequential steps: (1) identification of noise-related signal components, (2) linear regression to remove their influence, and (3) temporal band-pass filtering. The denoising model included the following nuisance regressors: five aCompCor components each from white matter and CSF time series (10 physiological noise regressors in total), 12 motion-related regressors (6 rigid-body motion estimates and their first-order temporal derivatives), and task-related effects modeled as event-related regressors convolved with the canonical hemodynamic response function, along with their first-order temporal derivatives. Outlier scans were identified using framewise displacement > 0.9 mm or global signal change > 5 STD via the ART toolbox. Each outlier timepoint (mean: 22 scans per subject out of 1,090 total scans) was entered as a separate nuisance regressor with a value of 1 at the corresponding scan and 0 elsewhere, allowing them to be regressed out independently during the denoising step. Task activation regression was performed to remove task-related variance from the BOLD signal, ensuring that functional connectivity estimates reflected condition-modulated interactions rather than shared task-evoked responses. After nuisance regression, a temporal band-pass filter (0.008–0.09 Hz) was applied to isolate low-frequency fluctuations. The effective degrees of freedom in the denoised time series ranged from 94.5 to 147.2 across subjects (mean: 143.1), reflecting the number of regressors and censored scans.

#### 2.6.3 ROI-to-ROI functional connectivity analysis

##### 2.6.3.1 First-level analysis

ROI-to-ROI connectivity (RRC) matrices were computed for each participant using the sets of ROIs derived from the task-based activation contrasts described in Section 2.6.1, comprising 47 ROIs from the dual-task cognitive-dominant contrast and 70 ROIs from the dual-task motor-dominant contrast. Task-based functional connectivity was estimated using a weighted ROI-to-ROI Connectivity (wRRC) measure (Nieto-Castanon, 2020), which quantifies condition-specific functional connectivity strength (i.e., functional connectivity during each cognitive or motor task, single and dual conditions) among the pre-defined set of ROIs (i.e., cognitive- and motor-dominant ROIs). wRRC matrices were computed using a weighted Least Squares (WLS) linear model with task-specific temporal weights identifying each individual task condition. In the present event-related design, task regressors were modeled as zero duration, time locked to the onset of the visual stimulus (cue) and convolved with the canonical hemodynamic response function. This approach captures transient, stimulus-evoked neural responses associated with task initiation while minimizing contributions from non-task-related fluctuations.

##### 2.6.3.2 Second-level analysis

Group-level analyses were performed using a General Linear Model framework implemented in CONN (Nieto-Castanon, 2020). For each ROI-to-ROI connection, a separate GLM was estimated, with first-level connectivity measures serving as dependent variables (one observation per subject per task condition), and age group as the between-subject factor. Because each subject contributed multiple connectivity measures across the five task conditions (Single Cognitive Go, Single Cognitive NoGo, Single Motor, Dual Go, Dual NoGo), group-level analyses accounted for the non-independence of these repeated measures by estimating the within-subject covariance structure across these five conditions. Connection-level hypotheses were then evaluated using multivariate parametric statistics with random-effects across subjects and correction for within-subject covariance when these five multiple measurements were present. Statistical inferences were conducted at the cluster level, where clusters represented groups of similar ROI-to-ROI connections. Cluster-level effects were evaluated using parametric statistics both within and between pairs of functional networks (Functional Network Connectivity; Jafri et al., 2008). Networks were defined using a complete-linkage hierarchical clustering procedure, which grouped ROIs according to a combination of anatomical proximity and functional similarity metrics (Nieto-Castanon, 2020). To control multiple comparisons, a two-step thresholding approach was applied: first, a connection-level threshold of *p* < 0.05 (uncorrected) was applied; second, cluster-level false discovery rate correction (*p*-FDR < 0.05) was used.

To localize age-related effects within brain regions associated with cognitive- and motor-dominant aspects of dual tasking, we conducted task domain-specific functional connectivity analyses separately for the cognitive-dominant and motor-dominant ROI sets defined in the activation contrasts (Dual Go - Single Motor, and Dual Go - Single Cognitive). Functional connectivity was assessed for each ROI set under both single-task and dual-task conditions.

##### 2.6.3.3 Data quality

Head motion was well controlled across participants. Only 6% of participants exhibited maximum head displacement exceeding 3 mm. This proportion is lower than previously reported rates of excessive motion during lower-limb motor tasks in the scanner (e.g., ~20% exceeding 5 mm, Reinhardt et al., 2020). After CONN denoising procedures, including scrubbing of outlier scans, the mean framewise displacement was 0.15 mm, which is comparable with values typically observed in resting-state functional connectivity studies (Parkes et al., 2018).

### 2.7 Multivariate brain–behavior association analysis

The final analysis step aimed to investigate multivariate associations between functional connectivity and dual-task behavioral performance, thereby providing complementary insights beyond univariate analyses. To identify these associations while mitigating multicollinearity and overfitting, we applied RGCCA using the mixOmics R package (version 6.22.0; Rohart et al., 2017).

RGCCA is a multiblock extension of classical canonical correlation analysis (CCA) that integrates multiple data blocks measured on the same set of individuals. The method extracts latent variables (components) from each block that maximize covariance across data blocks, while incorporating regularization to ensure stable estimation in high-dimensional settings. Subject-level component scores (projections of individuals onto latent components) were obtained from each RGCCA and examined separately by task-dominant network and age group. These components represent latent dimensions that capture shared variance across functional connectivity and behavioral measures, facilitating visualization and interpretation of multivariate brain–behavior relationships across task conditions.

To capture task domain-dominant effects, RGCCA were conducted separately for the cognitive-dominant network and motor-dominant network. Each analysis integrated three data blocks: (1) dual-task functional connectivity derived from the wRRC functional connectivity analysis, (2) single-task (cognitive and motor) functional connectivity derived from the same wRRC framework, and (3) dual-task behavioral measures, including mean RT, RT variability, error rate, and dual-task costs for both motor and cognitive modalities. In the RGCCA block design, the two brain blocks were fully connected to each other and to the behavioral block, allowing examination of multivariate associations between functional connectivity and behavior, as well as relationships between single-task and dual-task connectivity patterns within task-dominant network configurations.

To aid interpretation of the multivariate components, we additionally examined the variable loadings associated with Component 1 of the RGCCA models. Although loadings were examined for both models, only results from the motor-dominant network are reported in detail due to their stronger and more interpretable associations. These loading weights indicate the relative contribution of individual behavioral measures and functional connections to the latent brain–behavior dimension. Loadings were visualized to facilitate interpretation of the multivariate results (see Figs. 9 and 10).

In RGCCA, the regularization parameter tau controls covariance shrinkage for each block. Rather than relying on cross-validation, the RGCCA package uses the automatic shrinkage estimator proposed by Schäfer and Strimmer (2005), which analytically derives an optimal shrinkage intensity that balances bias and variance. Following Tenenhaus et al. (2017), tau was set to “optimal”, the default setting in RGCCA with τ estimated directly from sample data for each block. The approach is particularly advantageous when the number of variables exceeds the number of subjects, as in our study, where cross-validation can be unstable in small-sample behavioral datasets. Importantly, tau in RGCCA is not tuned for predictive accuracy but to stabilize covariance estimation for multiblock correlation analysis, making the Schäfer–Strimmer estimator a principled, reproducible, and efficient choice for this application.

## 3 Results

### 3.1 Behavioral results

Older and younger adults did not differ significantly in sex distribution or years of education. Participants also rated their general health and functional mobility using the SF-12 questionnaire (Ware et al., 1996). Among respondents, all participants reported their health as good, very good, or excellent. Two participants in each group did not complete the SF-12 questionnaire, resulting in missing values for these items. Functional mobility was assessed using SF-12 items evaluating limitations in moderate activities and stair climbing. The majority of participants reported no mobility limitations, and all participants were able to walk independently without assistive devices. Among older adults, 12.8% reported some limitations in moderate activities and 18.9% reported some limitations in stair climbing, whereas no such limitations were reported in younger adults.

As assessed by the TAP-M test battery, older adults showed significantly slower performance than younger adults in the distraction task, divided attention task (both auditory and visual modalities), and the Go/NoGo task. No significant group difference was observed in the executive control task. To control for multiple comparisons across the five cognitive variables, Bonferroni-adjusted *p*-values were calculated. Descriptive statistics and test results are shown in Table 1.

**Table 1.** Participant characteristics and TAP-M performance.

Measure	Older adults (n = 40)	Younger adults (n = 20)	*p* value
**Demographics**			
Age (years)	67.55 ± 7.09	28.00 ± 4.87	—
Gender (male %)	19 (47.5%)	11 (55.0%)	0.784
Education (years)	13.2 ± 3.09	14.6 ± 1.53	0.161
**SF-12 health status**			
Self-rated health (excellent/very good/good), n (%)*	38/38 (100%)	18/18 (100%)	—
Moderate activity limitation, n (%)	5 (12.8%)	0 (0%)	—
Stair climbing limitation, n (%)	7 (18.9%)	0 (0%)	—
**TAP-M (reaction time, ms)**			
Distraction	490.7 ± 9.78	414.8 ± 11.34	**<0.001**
Executive control	662.2 ± 16.99	623.1 ± 13.83	0.396
Divided attention-auditory	651.7 ± 18.60	579.4 ± 14.51	**0.017**
Divided attention-visual	854.8 ± 20.12	734.2 ± 13.48	**<0.001**
Go/NoGo	448.2 ± 10.08	388.2 ± 29.17	**<0.001**

Note: Values are mean ± SE unless otherwise indicated. Bold values in the TAP-M section indicate statistically significant group differences after Bonferroni correction (*p* < 0.05).

* Self-rated health refers to the first SF-12 item. Percentages are calculated among respondents.

SF-12 = Short Form-12 Health Survey; TAP-M = Test of Attentional Performance.

To address our primary research question, we examined RTs from the in-scanner dual-task paradigm to assess how dual-task demands influenced motor and cognitive performance in younger and older adults. RTs differed significantly between younger and older adults (main effect age group: *F*(1, 52) = 4.22, *p* = 0.045, η²_G_ = 0.044), between single and dual-task conditions (main effect task: *F*(1, 52) = 275.10, *p* < 0.001, η²_G_ = 0.303), and between cognitive and motor task domain (main effect of domain: *F*(1, 52) = 173.15, *p* < 0.001, η²_G_ = 0.433). No significant two-way interactions were observed; however, a significant three-way interaction between age, task, and domain was found (*F*(1, 52) = 11.79, *p* = 0.001, η²_G_ = 0.027), indicating that dual-task demands affected cognitive and motor performance differently across age groups (see Table 2). To provide a more transparent representation of individual variability, Figure 2 displays subject-level data points and trajectories in addition to group means.

**Table 2.** Mean reaction time (RT), RT variability (SD), and error rate are reported for younger and older adults across single- and dual-task conditions and motor and cognitive domains, including derived dual-task costs (16 outcome measures in total).

		Domain					
		Cognitive task			Motor task		
Task	Measure	Old	Young	*p*	Old	Young	*p*
Single	Mean RT (s)	0.535 ± 0.014	0.459 ± 0.014	**3.1e-4**	0.324 ± 0.011	0.319 ± 0.016	0.827
Dual	Mean RT (s)	0.617 ± 0.016	0.599 ± 0.024	0.528	0.496 ± 0.018	0.426 ± 0.025	0.030
Dual-task cost (%)		15.8 ± 2.69	31.4 ± 4.96	0.009	55.3 ± 4.57	33.9 ± 4.36	**0.001**
Single	Variability (s)	0.085 ± 0.004	0.083 ± 0.005	0.690	0.112 ± 0.004	0.107 ± 0.006	0.539
Dual	Variability (s)	0.128 ± 0.005	0.113 ± 0.006	0.075	0.168 ± 0.012	0.111 ± 0.005	**5.5e-5**
Dual-task cost (%)		56.2 ± 7.87	44.3 ± 13.3	0.447	55.3 ± 11.6	8.4 ± 6.70	**9.2e-4**
Single	Error rate (%)	2.58 ± 0.61	5.28 ± 1.11	0.038	0.10 ± 0.05	0.00 ± 0.00	0.044
Dual	Error rate (%)	3.83 ± 0.82	6.30 ± 1.39	0.134	4.24 ± 1.25	0.22 ± 0.10	**0.0026**

Two-sample t-tests were used to compare groups for each outcome measure. Values represent mean ± standard error (SE).

Bold *p*-values indicate significance at Bonferroni-corrected threshold (*p* < 0.0031).

For RT variability, all three main effects were significant: age (*F*(1, 52) = 7.27, *p* = 0.009, η²_G_ = 0.058), task (F(1, 52) = 37.10, *p* < 0.001, η²_G_ = 0.153), and domain (*F*(1, 52) = 27.12, *p* < 0.001, η²_G_ = 0.071). In addition, we observed significant two-way interactions between age and task (*F*(1, 52) = 8.55, *p* = 0.005, η²_G_ = 0.040) and between age and domain (*F*(1, 52) = 7.79, *p* = 0.007, η²_G_ = 0.022), whereas the task × domain interaction was not significant. Crucially, the three-way interaction of age group, task condition, and domain was also significant (*F*(1, 52) = 6.59, *p* = 0.013, η²_G_ = 0.020), indicating that performance variability increased most strongly in older adults during motor dual tasking, whereas variability in the cognitive domain remained largely comparable across groups (see Table 2). A similar pattern was observed for error rates, with greater dual-task-related increase in the motor task in older adults (see Table 2). Note that a post hoc analysis of pedaling cycle time and its variability yielded a comparable pattern (Supplementary Table S2). While mean cycle time remained largely constrained by the fixed task structure, variability increased under dual-task conditions, with a more pronounced effect in older adults.

**Fig. 3.**
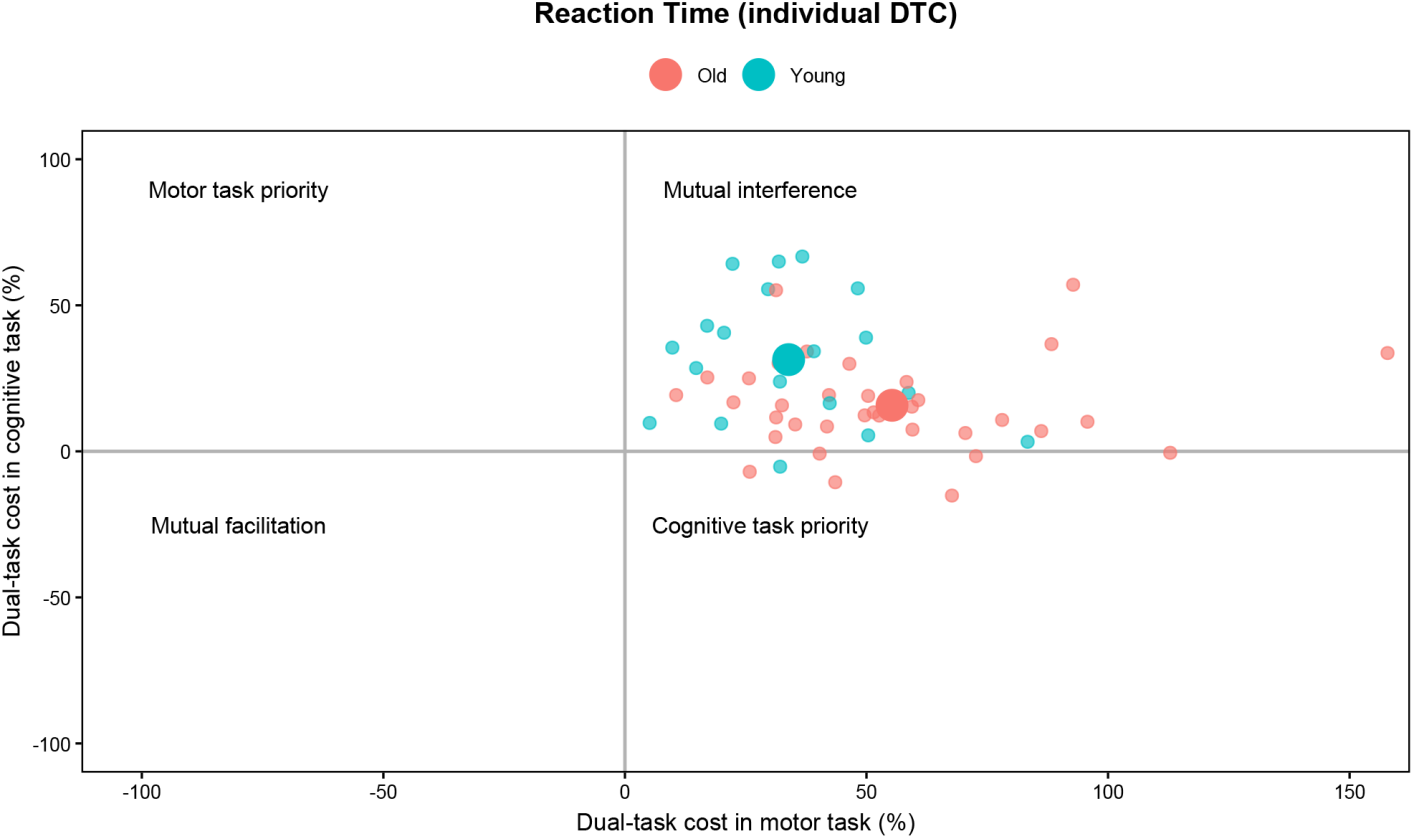
Individual-level dual-task costs across cognitive and motor reaction times. Dual-task cost (%) in the motor task domain is plotted on the x-axis and dual-task cost (%) in the cognitive task domain on the y-axis, allowing classification into mutual interference, motor priority, cognitive priority, or mutual facilitation. Red circles indicate individual older adults, and blue circles indicate individual younger adults (group means denoted by larger circles). Due to missing behavioral data, six participants (Older: five, Younger: one) were excluded from this analysis. Note that dual-task interference was present in both domains and in both age groups. However, the distribution of interference differed by age: younger adults tended to exhibit larger cognitive DTCs, whereas older adults showed disproportionately greater motor DTCs.

To further characterize inter-individual variability in dual-task performance, we additionally visualized individual-level dual-task costs (DTCs) across cognitive and motor task domains (Fig. 3). Plotting motor DTC against cognitive DTC allowed classification of performance patterns into motor task priority, cognitive task priority, mutual interference, or facilitation. Dual-task interference was evident in both task domains across age groups. Notably, younger adults tended to exhibit relatively larger cognitive DTCs, whereas older adults showed comparatively greater motor DTCs.

### 3.2 Cognitive-dominant and motor-dominant components during dual tasking (ROI definition)

**Fig. 4.**
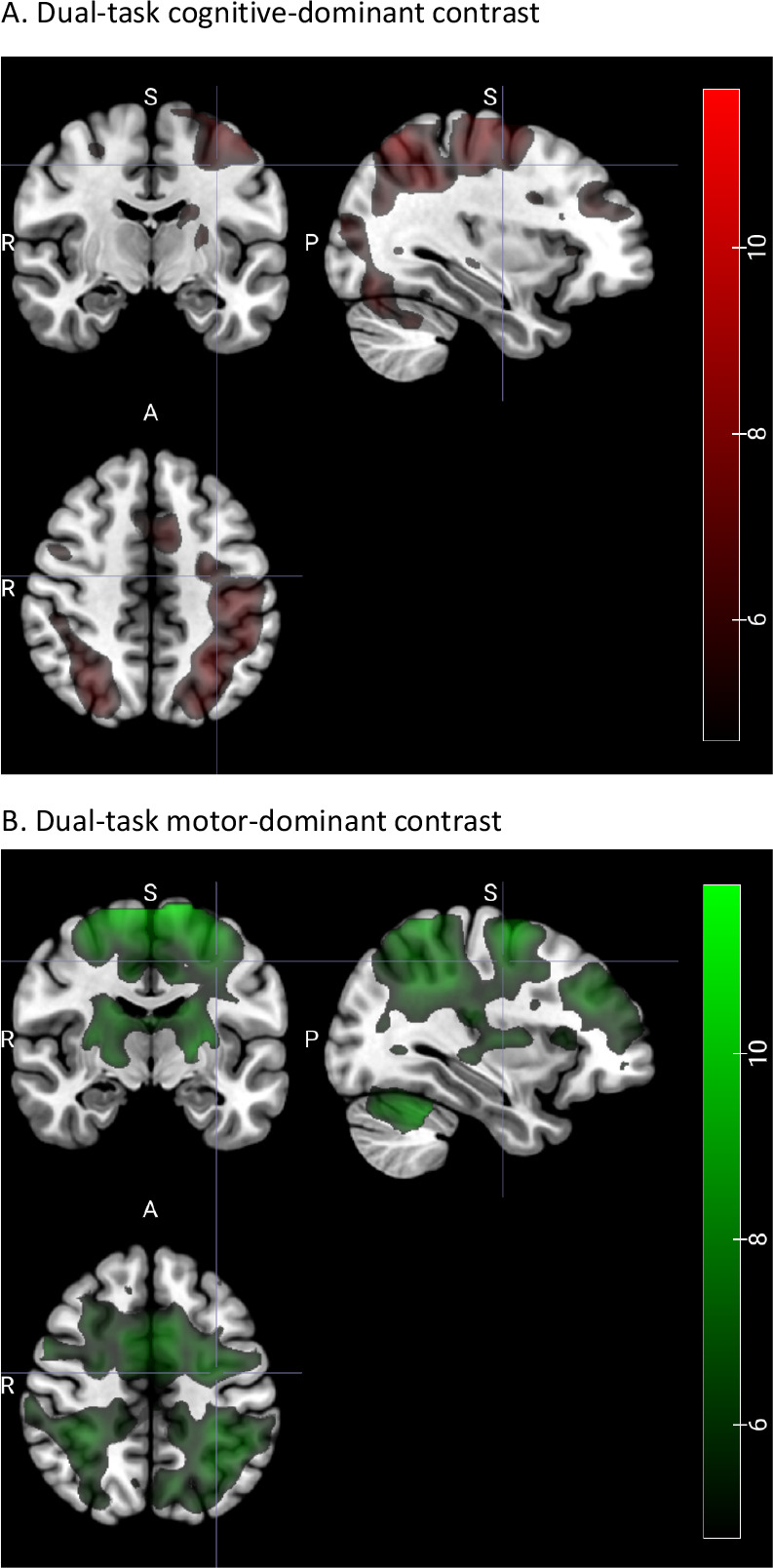
Brain regions identified from contrasts emphasizing the cognitive-dominant and motor-dominant components of dual tasking, used for ROI definition in the connectivity analyses. Shown are group-level whole-brain activation patterns for (A) the dual-task cognitive-dominant contrast (Dual Go – Single Motor) and (B) the dual-task motor-dominant contrast (Dual Go – Single Cognitive Go), across age groups, thresholded at *p* < 0.05 (FWE-corrected).

To identify regions associated with the cognitive- and motor-dominant aspects of dual-task performance for subsequent connectivity analyses, we used two targeted contrasts defined at the first level: the dual-task cognitive-dominant contrast (Dual Go – Single Motor) and the dual-task motor-dominant contrast (Dual Go – Single Cognitive Go). The dual-task cognitive-dominant contrast revealed activation primarily in prefrontal and parietal areas, as well as occipital cortices (Fig. 4A), whereas the dual-task motor-dominant contrast was associated with activity in bilateral precentral and postcentral gyri, superior parietal lobule, basal ganglia, and cerebellum (Fig. 4B). While these patterns partly overlap, as expected given the integrative nature of dual tasking, they nevertheless enabled the definition of ROI masks and the separate examination of connectivity within networks more strongly associated with either cognitive- or the motor-dominant processing.

### 3.3 Age-related differences in functional connectivity within the dual-task cognitive-dominant and motor-dominant networks

**Fig. 5.**
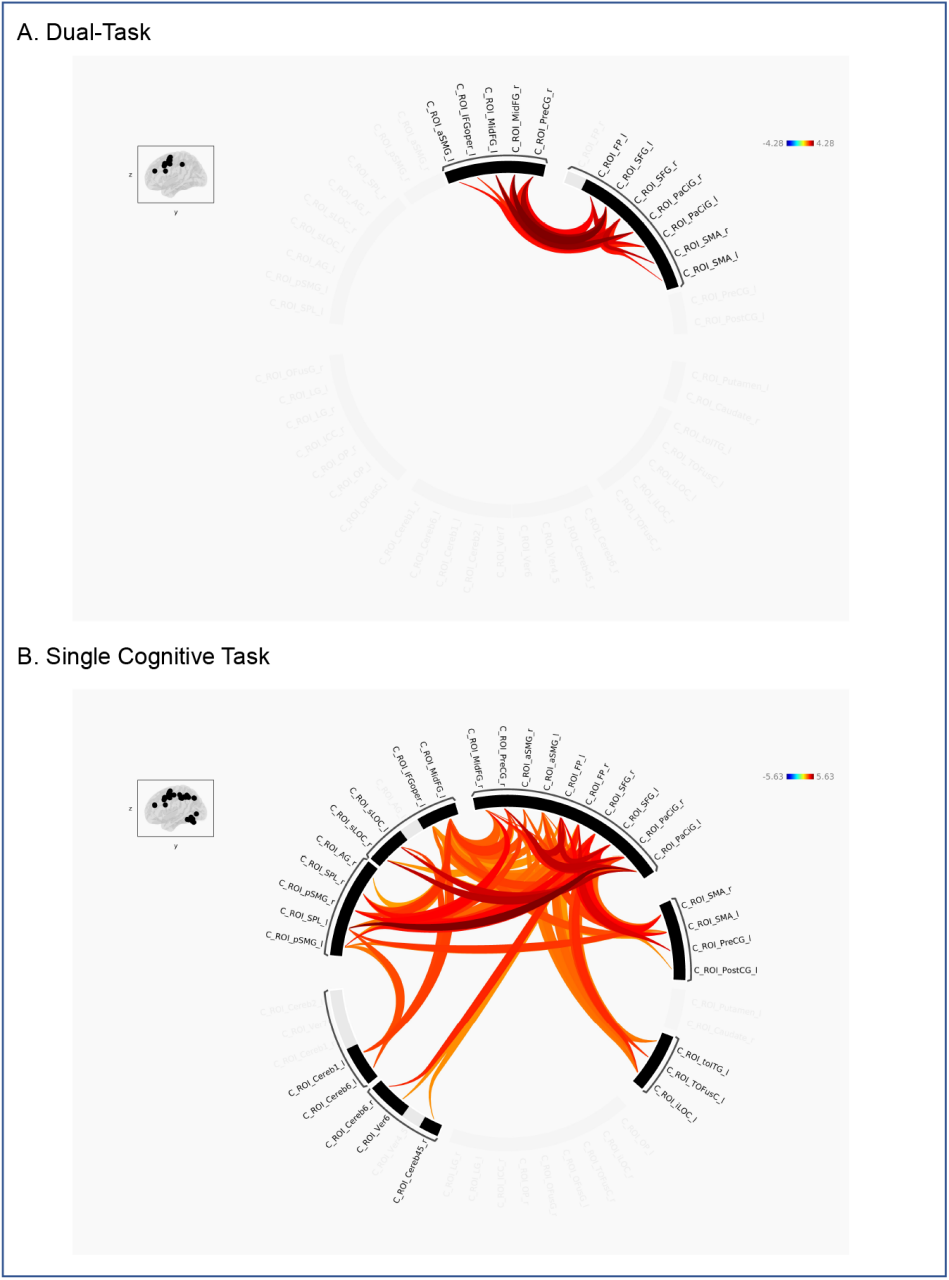
Group-level ROI-to-ROI functional connectivity within the cognitive-dominant network. Shown are age-group differences for (A) the dual-task condition and (B) the single cognitive task. Significant effects (Older > Younger; *p* < 0.05, FDR corrected at the cluster level) are visualized as circular graphs, with color gradients representing *t*-values (warmer colors indicate stronger connectivity in older adults; cooler colors indicate stronger connectivity in younger adults). Corresponding matrix plots are shown in Supplementary Figure S2.

**Fig. 6.**
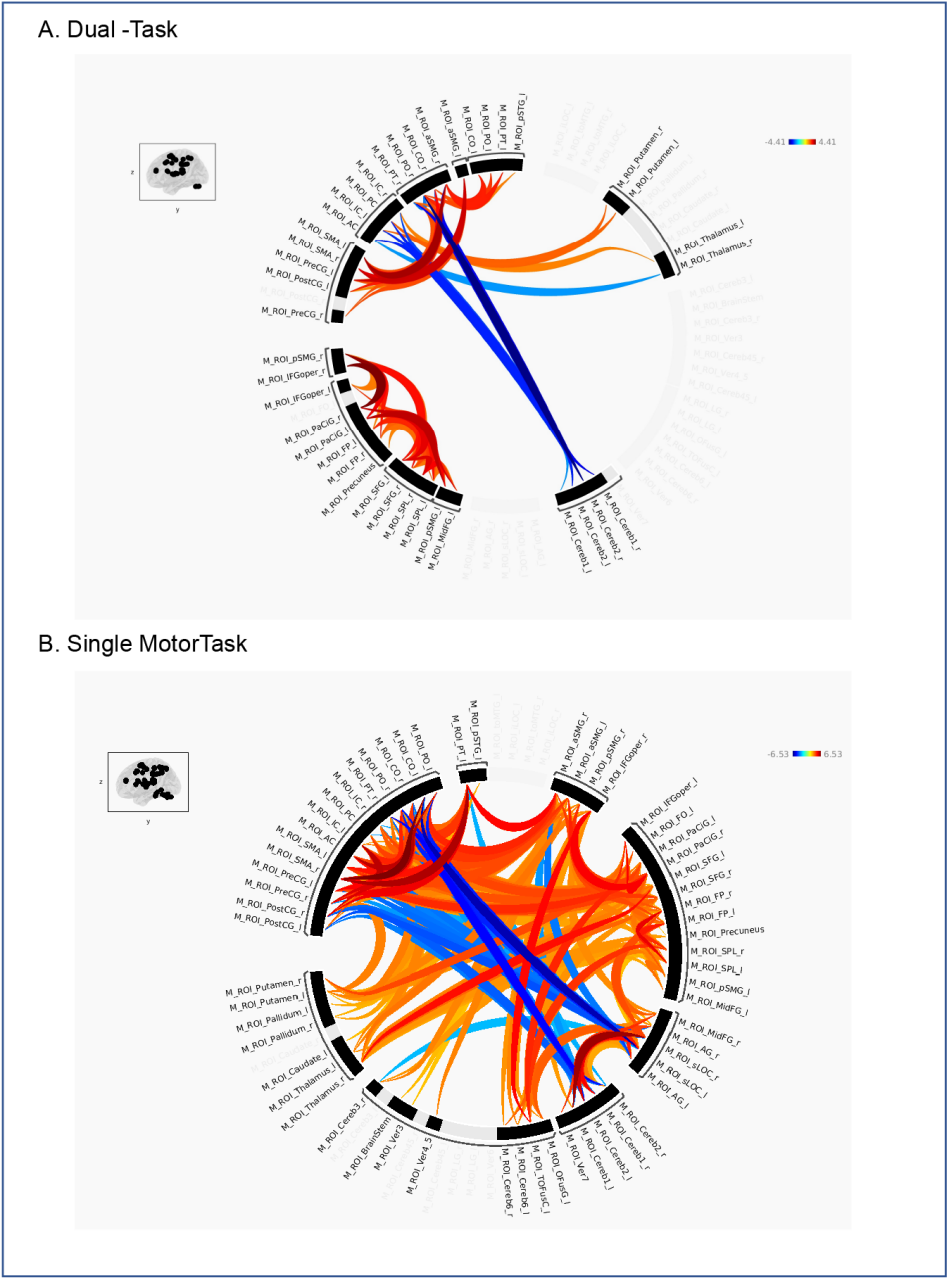
Group-level ROI-to-ROI functional connectivity within the motor-dominant network. Shown are age-group differences for (A) the dual-task condition, and (B) the single motor task. Significant effects (Older > Younger; *p* < 0.05, FDR corrected at the cluster level) are visualized as circular graphs, with color gradients representing *t*-values (warmer colors indicate stronger connectivity in older adults; cooler colors indicate stronger connectivity in younger adults). Corresponding matrix plots are shown in Supplementary Figure S3.

Building on the behavioral evidence for age-related differences in dual-task costs for motor and cognitive performance, we next examined whether functional connectivity showed corresponding differences within the dual-task cognitive- and motor-dominant networks. Given that age-related effects were already evident in the cognitive-dominant domain under single-task conditions, connectivity analyses were conducted separately for each task (dual task and the respective single task) and task domain (cognitive- and motor-dominant within the ROIs shown in Fig. 5 and Fig. 6). This approach allowed us to examine whether age-related reorganization of network interactions emerges primarily under dual-task demands or is already present during single-task conditions, and whether such effects are more pronounced in cognitive-dominant or motor-dominant networks.

Within the cognitive-dominant network, dual tasking was associated with a relatively limited set of connections that were significantly stronger in older than in younger adults (Fig. 5A). These involved frontal executive regions such as bilateral superior frontal gyrus (SFG), left frontal pole (FP_l), bilateral middle frontal gyrus (MidFG), as well as motor-planning areas such as the supplementary motor area (SMA) and right precentral gyrus (PreCG_r). Additional involvement of the paracingulate cortex (PaCiG) and left anterior supramarginal gyrus (aSMG_l) was also observed. In contrast, during the single cognitive task, more widespread increases in connectivity in older adults were observed (Fig. 5B), encompassing bilateral frontal and parietal areas including the superior and middle frontal gyri, supramarginal gyrus (SMG), and superior parietal lobules (SPL), as well as several cerebellar clusters. Notably, the extent of connectivity differences was reduced under dual-task conditions compared with the single-task condition.

Within the motor-dominant network, dual tasking was associated with a broader and more distributed pattern of age-related differences in task-related connectivity (Fig. 6A). These effects extended beyond frontal and parietal regions to include posterior cortical areas (e.g., precuneus, occipital cortex), subcortical structures (thalamus, putamen), and the cerebellum. The connectivity profile comprised both increases (Old > Young) and decreases (Old < Young) in connectivity. Positive effects were primarily observed within frontal and parietal ROIs, indicating increased connectivity in older adults, whereas negative effects were concentrated in posterior and subcortical regions—particularly between the cerebellum and parietal cortex—indicating reduced coupling within posterior sensorimotor and cerebellar pathways.

During the single motor task, age-related effects in functional connectivity were extensive, encompassing both cortical and subcortical regions (Fig. 6B). Older adults exhibited increased functional connectivity across extended bilateral frontal and sensorimotor regions, including the precentral and postcentral gyri, SMA, and basal ganglia. At the same time, reductions in connectivity were observed between occipital, posterior parietal, and cerebellar regions. These findings suggest that even during a relatively simple motor task, aging is associated with widespread network alterations.

Taken together, we observed distinct age-related connectivity changes in both motor-dominant and cognitive-dominant networks, which were evident not only under dual-task demands but also during single-task conditions. While the present analyses focused on characterizing age-related connectivity changes within these task-dominant networks, we additionally report, for completeness, connectivity results derived from dual-task–specific regions (Dual Task – [Single Motor + Single Cognitive]), abbreviated as DT − (SM + SC), and compared it with the Dual Task − Single Cognitive (DT – SC) and Single Motor (SM) contrasts in the Supplementary Figure S4. The DT − (SM + SC) contrast yields a connectivity pattern that is selectively expressed during the dual-task condition and largely absent during single motor and single cognitive conditions, consistent with the engagement of dual-task–specific integrative control processes.

### 3.4 Linking functional connectivity with behavioral performance

**Fig. 7.**
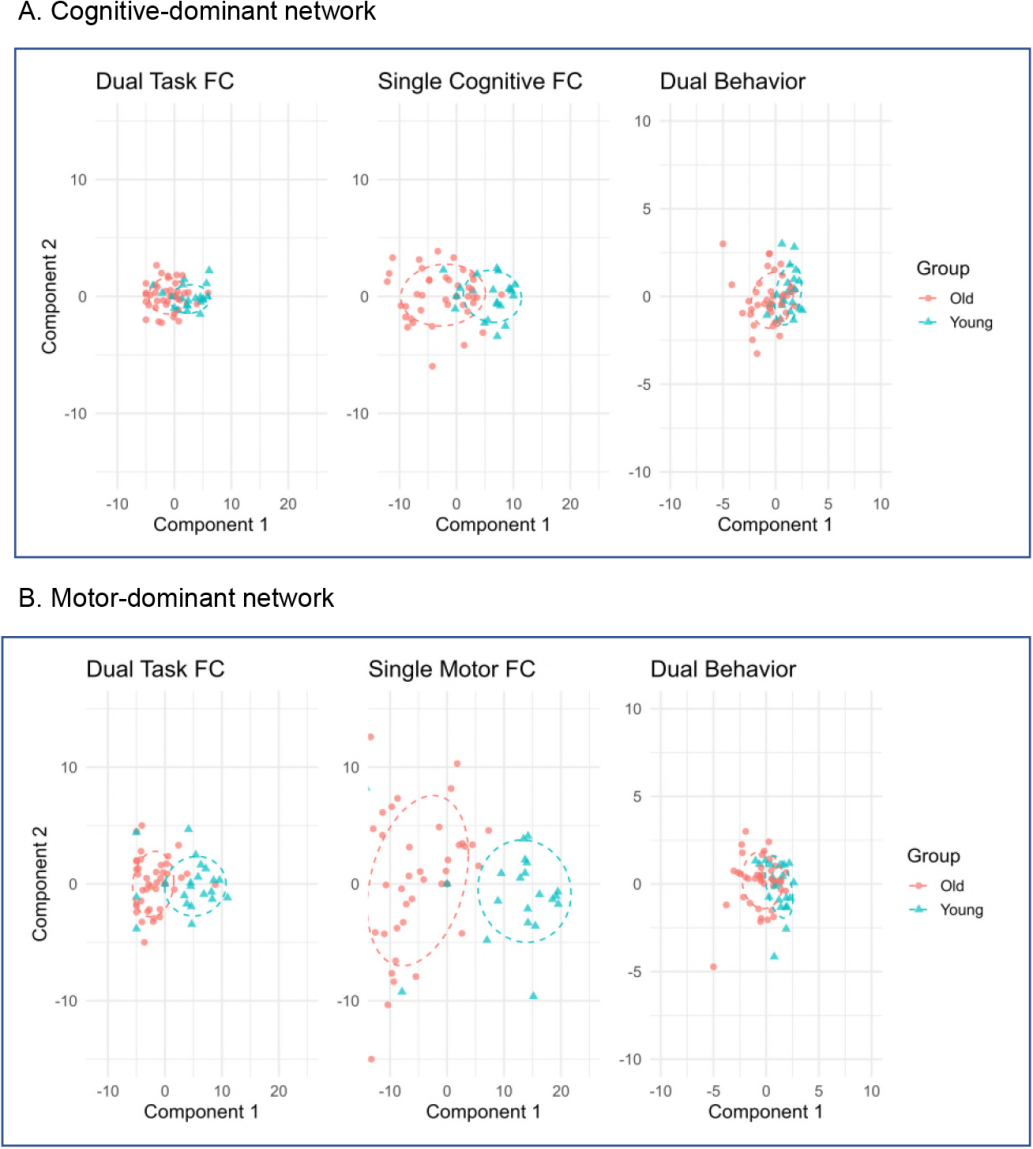
Multivariate component scores from RGCCA by task-dominant network and age groups. Panel (A) displays component scores within the cognitive-dominant network, integrating dual-task functional connectivity, single-task cognitive functional connectivity, and dual-task behavioral performance (Dual-Task FC, Single Cognitive FC, Dual-Task Behavior). Panel (B) shows corresponding component scores within the motor-dominant network, integrating dual-task functional connectivity, single-task motor functional connectivity, and dual-task behavioral performance (Dual-Task FC, Single Motor FC, and Dual-Task Behavior). Each plot displays Component 1 versus Component 2 for younger adults (blue triangles) and older adults (red circles), with 68% confidence ellipses indicating group dispersion. Separation along Component 1 reflects age-related differences, whereas increased overlap indicates reduced multivariate separation between groups. For better visual comparison, the same data are also plotted with the axis limits tailored to each data block in Supplementary Figure S5. FC: functional connectivity.

To investigate how individual differences in task-based functional connectivity relate to behavioral performance, we conducted multivariate brain–behavior analyses using RGCCA. This approach integrated connectivity measures from both dual- and single-task conditions with a predefined set of behavioral performance measures collected under dual-task conditions (see Section 2.7). Analyses were performed across the combined sample of younger and older adults, focusing on shared dimensions of covariation between functional connectivity and behavioral measures. Figure 7 shows subject-level component scores derived from the RGCCA, separated by task network and age groups. These components represent latent dimensions that maximize shared variance across functional connectivity and behavioral blocks, enabling visualization of group differences in multivariate brain–behavior associations.

The upper panel (Fig. 7A) shows results for the cognitive-dominant network, integrating dual-task functional connectivity, single cognitive task functional connectivity, and dual-task behavioral performance. The lower panel (Fig. 7B) presents corresponding results for the motor-dominant network, incorporating dual-task functional connectivity, single motor task functional connectivity and dual-task behavior. Participant scores are projected onto the first two RGCCA components, revealing distinct patterns across the two networks. In the motor-dominant network (Fig. 7B), subject scores on Component 1 showed a clear tendency for age-related separation: older adults clustered toward the lower end, whereas younger adults were distributed across a broader and higher range. In contrast, the cognitive-dominant network (Fig. 7A) exhibits a more compressed distribution along both components. Although group differences remained visible along Component 1, the separation was less pronounced, with confidence ellipses of the two age groups showing greater overlap, particularly for dual-task functional connectivity and dual-task behavior. This pattern reflects a reduced multivariate separation between age groups in cognitive-related functional connectivity and dual-task behavior.

**Fig. 8.**
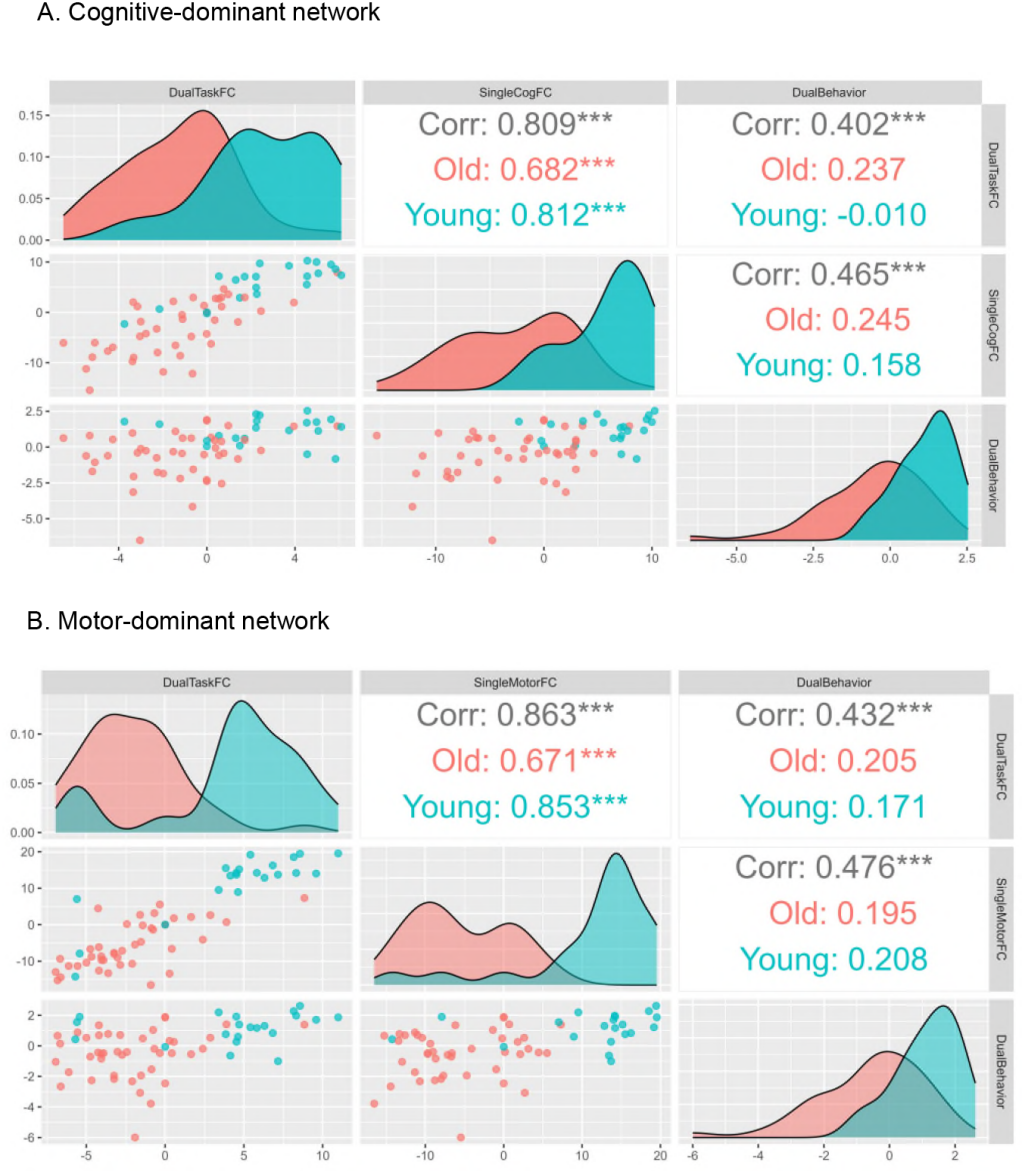
Pairwise correlations for Component 1 scores from the RGCCA for (A) the cognitive-dominant network and (B) the motor-dominant network. Scatterplots (lower panels) and density plots (diagonal panels) show distributions and relationships among dual-task functional connectivity, single-task functional connectivity, and dual-task behavioral performance (Dual-Task FC, Single-Task FC, and Dual-Task Behavior). Overall and age-group-specific correlations (older adults in red, younger adults in blue) are reported in the upper panels. Asterisks indicate significance levels (***p < 0.001). FC: functional connectivity.

Because component 1 showed greater variability and clearer age-related structure across groups, subsequent correlation analyses focused on this component. As shown in Figure 8, both motor- and cognitive-dominant networks exhibited significant associations between dual-task performance and functional connectivity under both dual-task and single-task conditions. Compared with younger adults, older adults tended to show slightly stronger correlations between functional connectivity and dual-task performance in both networks, except for the coupling between single-task motor functional connectivity and dual-task performance. However, the coupling between single- and dual-task functional connectivity was weaker in older adults than in younger adults. Notably, functional connectivity during the single-task condition correlated with behavior as strongly as connectivity during the dual-task condition (e.g., Motor: *r* = 0.476 vs. 0.432; Cognitive: *r* = 0.465 vs. 0.402), suggesting that connectivity during simpler tasks captures task-relevant functional organization beyond baseline activity. Diagonal density plots further indicated partial overlap between age groups in the dual-task cognitive-dominant network, whereas the motor-dominant network showed clearer age-related separation.

**Fig. 9.**
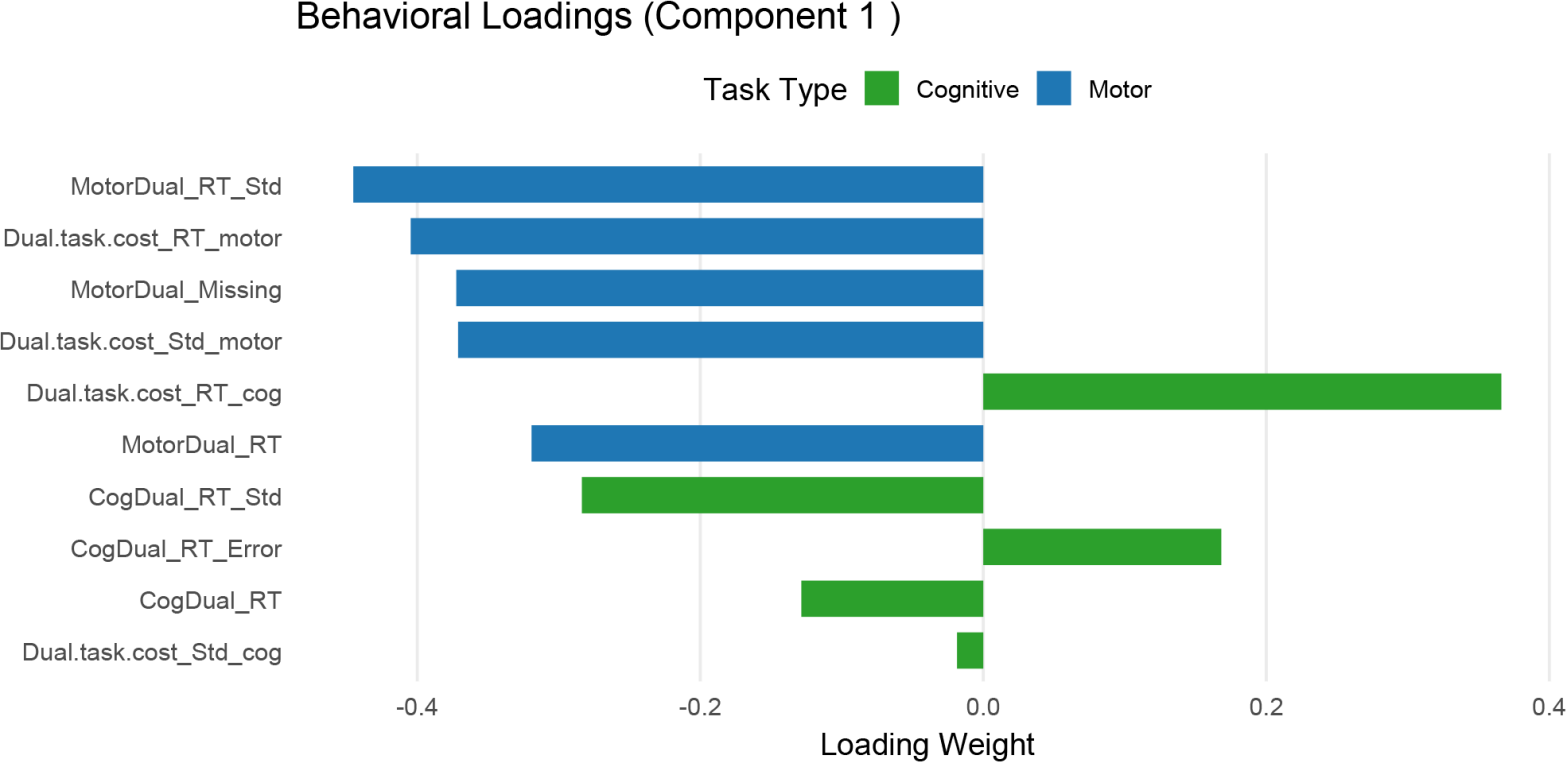
Behavioral loadings for Component 1 from the RGCCA model (motor-dominant network). Bar plots show the contribution of dual-task behavioral measures to the latent component identified by the RGCCA. Negative and positive values indicate the direction and magnitude of each variable’s contribution to the component. Measures of response time variability under dual-task conditions (e.g., MotorDual_RT_Std) show the largest loading, with additional contributions from motor dual-task cost in mean RT (Dual.task.cost_RT_motor) and other variability-related measures.

**Fig. 10.**
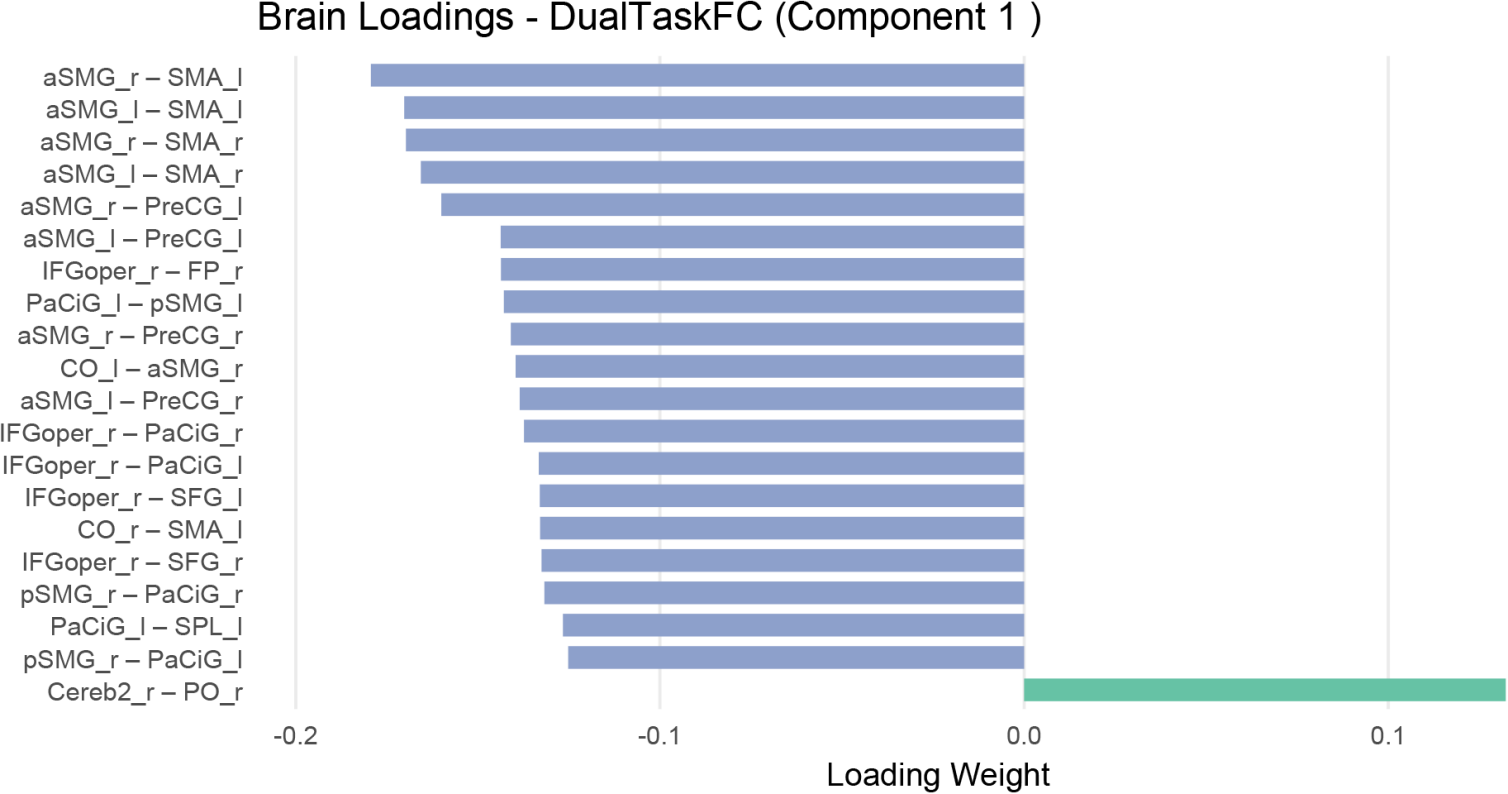
Brain connectivity loadings for Component 1 from the RGCCA model (motor-dominant network). Bar plots show the functional connections contributing to the same latent brain–behavior component shown in Figure 9. For visualization, the top 20 connections with the largest absolute loading weights are shown. Loading weights indicate the relative contribution of each connection to the multivariate brain–behavior association. Prominent loadings involve connections linking parietal and frontal motor-control regions, including the anterior supramarginal gyrus (aSMG), supplementary motor area (SMA), and precentral gyrus (PreCG).

To further characterize the multivariate brain–behavior associations identified by the RGCCA, we examined the variable loadings contributing to Component 1 of the motor-dominant network (Figs. 9–10). This analysis focused on the motor-dominant network because behavioral results showed that response time variability increased most strongly during motor dual tasking in older adults (Section 3.1). Behavioral loadings indicated that dual-task motor RT variability (MotorDual_RT_Std) carried the largest contribution to the latent component, with additional strong contributions from motor dual-task cost in mean RT (Dual.task.cost_RT_motor) and several other variability-related measures (Fig. 9). Examination of the corresponding brain connectivity loadings revealed prominent contributions from connections linking parietal and frontal motor-control regions, particularly the anterior supramarginal gyrus (aSMG), supplementary motor area (SMA), and precentral gyrus (PreCG) (Fig. 10), suggesting that this behavioral dimension is associated with coordinated interactions within a fronto-parietal motor-related network.

## 4 Discussion

In this study, we investigated task-based functional connectivity during cognitive–motor dual tasking in older and younger adults using an MRI-compatible pedaling paradigm. Three main findings emerged. First, older adults showed preserved cognitive dual-task performance but greater dual-task costs in motor reaction times and variability. Second, age-related differences in functional connectivity were evident not only under dual-task demands but also during the respective single tasks. These effects were network specific: cognitive-dominant networks showed relatively selective connectivity changes involving frontal executive and motor-planning circuits, whereas motor-dominant networks showed broader system-wide reorganization, including reduced coupling among posterior, subcortical, and cerebellar sensorimotor regions. Third, multivariate RGCCA revealed systematic age-related differences in latent connectivity patterns across both single- and dual-task conditions that aligned with a latent behavioral dimension.

### 4.1 Cognitive dual-task effects in aging

Behaviorally, we observed a significant increase in RT for the single cognitive Go/NoGo task in the older group, but no significant age-group differences in mean RT, RT variability, or error rate during the dual-task condition. The longer single-task RT in older adults likely reflects age-related baseline slowing in processing speed, a widely documented feature of healthy aging (Ji et al., 2019; Salthouse, 1996; Verhaeghen & Salthouse, 1997). However, processing speed does not fully account for all age-related cognitive differences (Anstey et al., 2001), indicating that baseline slowing represents only one component within a broader reorganization of cognitive and neural systems (Harada et al., 2013). In the present sample, baseline slowing occurred alongside preserved accuracy and intact executive control (consistent with Baudouin et al. (2019) and our TAP-M findings), suggesting reduced processing efficiency rather than generalized cognitive decline. This baseline difference has important implications for interpreting dual-task effects. Dual-task cost indexes the incremental performance change from single- to dual-task conditions and is, therefore, inherently dependent on the baseline performance, such that slower baseline responses can attenuate proportional cost estimates. Older adults responded more slowly but more accurately in the single cognitive task, consistent with a more conservative speed–accuracy trade-off (Furstenberg et al., 2019; Lucci et al., 2013). Under such a cautious baseline strategy, the proportional increase in RT under dual-task conditions may be attenuated—not because interference is absent, but because performance already operates at a slower and more conservative baseline level. Accordingly, when expressed as proportional dual-task cost, cognitive performance was affected in both age groups, but the magnitude of the effect was smaller in older adults. In this sense, baseline processing-speed differences and dual-task interference represent dissociable components of performance that may differentially contribute to age-related effects. This interpretation aligns with dual-task frameworks suggesting that reduced processing speed can coexist with relatively preserved time-sharing capacity in healthy aging, depending on task demands (Tellinghuisen et al., 1996; Wickens et al., 1987).

Consistent with this dissociation, we observed clear age differences in single-task cognitive RT, but no disproportionate cognitive DTC in older adults relative to younger adults. Comparable patterns have been reported by O’Connell and Basak (2018) and by Plummer-D’Amato et al. (2012) across two different cognitive tasks. Notably, Plummer-D’Amato et al. (2012) demonstrated that lower education was associated with greater cognitive dual-task costs, while gait performance remained unaffected. In the present study, both age groups had relatively high and comparable years of education, which may partly account for the preserved cognitive performance under dual-task conditions. This lack of group differences may also reflect the relative ease of the Go/NoGo paradigm employed here, potentially reducing sensitivity to age-related decline. This interpretation aligns with previous dual-task findings showing that the complexity of the cognitive component critically modulates the magnitude of age effects (Bianchini et al., 2022; Goh et al., 2021). Together, these findings indicate that cognitive performance was largely preserved under dual-task conditions in older adults, despite clear baseline slowing. Although a ceiling effect in cognitive reaction time cannot be fully excluded, the absence of compression in RT distributions and the presence of robust dual-task effects in motor variability suggest that the reduced cognitive dual-task effect more likely reflects strategic prioritization rather than limited measurement sensitivity. This preservation, however, raises the possibility that dual-task costs may be redistributed to other domains, particularly the motor-dominant system, when cognitive and motor demands must be managed concurrently.

### 4.2 Motor dual-task effects in aging

In contrast to the preserved cognitive performance under dual-task conditions in older adults, the motor task domain showed pronounced age-related dual-task effects. In the single motor task, mean RT did not differ between age groups, suggesting comparable motor response timing under the externally imposed rhythmic pacing of the task. Because this rhythm was fixed and substantially slower than overground walking cadence, motor responses were highly predictable and required minimal trial-to-trial response selection, which likely reduced age-related differences in mean RT under single-task conditions.

By contrast, under dual-task conditions, older adults showed significantly higher dual-task costs across motor RT, variability, and error rate, with motor dual-task variability showing the largest effect. This dissociation suggests that aging primarily affects the stability and consistency of motor responses when cognitive and motor demands must be coordinated concurrently. Motor automaticity provides a useful framework for interpreting this pattern of dual-task interference (Wu et al., 2015). Automatic motor behaviors typically require minimal attentional resources (Passingham, 1996) and, therefore, typically show greater resistance to interference when performed concurrently with a cognitive task. The pronounced increase in motor response variability observed in older adults during dual tasking, therefore, suggests reduced motor automaticity, indicating greater reliance on attentional control to maintain stable motor output.

Although the pedaling task was externally paced and, therefore, differed from fully self-paced gait, the substantial dual-task costs observed indicate that the paradigm remained sensitive to age-related differences in the ability to sustain stable motor performance under divided attention. Independent TAP-M assessment provides converging evidence for interpreting these behavioral findings. The TAP-M results revealed a selective pattern of age-related attentional changes rather than a generalized cognitive decline. While executive control was preserved, older adults showed pronounced slowing in tasks emphasizing processing speed (Distraction, Go/NoGo) and reduced performance in divided attention, particularly in the visual modality. The strongest deficit was observed in visual divided attention, suggesting a specific age-related vulnerability in allocating attentional resources across competing visual streams. During scanning, participants were required to concurrently process visual information related to both the cognitive task (Go/NoGo stimuli) and the motor task (pedaling cues), increasing demands on divided attention. Reduced divided-attention capacity may, therefore, represent a key cognitive bottleneck that constrains the stability of motor output under dual-task conditions (Fakorede et al., 2025; Sun & Shea, 2016).

A similar pattern of disproportionate motor decrements under dual-task conditions has been reported in both fMRI studies using pedaling paradigms (Bürki et al., 2017) and ground-walking studies (Hollman et al., 2007; Piche et al., 2023). The predominantly motor decrement observed here also aligns with a recent systematic review and meta-analysis of 39 studies on locomotor-cognitive dual tasking (Mustafovska et al., 2025), which reported that variability in walking velocity during overground walking showed the largest age-related group difference. Neuroimaging studies of mobility further demonstrate that increased cognitive demands during locomotor tasks elicit greater prefrontal recruitment in older adults, reflecting heightened reliance on top–down motor control mechanisms (Holtzer et al., 2014). Accordingly, our findings should not be interpreted as evidence of isolated motor decline, but rather as reflecting increased interdependence between cognitive and sensorimotor systems (Huxhold et al., 2009; Li et al., 2018; Li & Lindenberger, 2002). Under dual-task conditions, this intensified cross-domain coupling may render motor stability more vulnerable to interference. In this context, the disproportionate increase in motor variability observed in older adults likely reflects greater dependence of motor performance on central attentional capacity rather than reflecting isolated motor dysfunction (Künstler et al., 2018).

### 4.3 Functional network-level reorganization in cognitive- and motor-dominant networks

Consistent with behavioral findings, our neuroimaging analyses revealed distinct functional connectivity patterns during single- and dual-task processing in younger and older adults. In the cognitive-dominant network, older adults showed widespread strengthened (Old > Young) connectivity under single-task conditions, predominantly involving bilateral frontal and parietal regions, such as the superior and middle frontal gyri, SMG, SPL, and cerebellar clusters. These effects were largely positive, indicating increased functional coupling within the cognitive control network in older adults than younger adults. These findings are in line with prior task-based connectivity studies showing that aging is associated with reorganization of large-scale networks, often involving increased frontal network recruitment during cognitive engagement (Geerligs et al., 2014; Matthäus et al., 2012; O’Connell & Basak, 2018; Stojan et al., 2023). Notably, Geerligs et al. (2014) demonstrated that such increased recruitment in older adults can emerge even during relatively simple tasks, consistent with the present findings. Under dual-task conditions, strengthened (Old > Young) connectivity was more focused and largely confined to frontal executive regions (e.g., bilateral SFG, left FP, bilateral MidFG) and motor-planning regions (e.g., SMA and precentral gyrus). This reduction in the spatial extent of connectivity differences compared with the single-task condition suggests a shift from widespread baseline differences toward more focused, task-dependent network configurations. This pattern suggests a targeted upregulation of frontally mediated cognitive control resources, consistent with compensatory recruitment to manage increased task demands (Cappell et al., 2010; Droby et al., 2022; Matthäus et al., 2012).

Behaviorally, this pattern may help explain why older adults were able to maintain cognitive performance under dual-task conditions at levels comparable with younger adults. Although the compensation-related utilization of neural circuits hypothesis (CRUNCH) and the scaffolding theory of aging and cognition (STAC) were originally formulated in terms of regional activation (Cabeza et al., 2018; Park & Reuter-Lorenz, 2009), our findings are consistent with their extension to the connectivity level, where compensation may be expressed not only through local overactivation but also through strengthened inter-regional coupling (Grady et al., 2016; Penhale et al., 2024). The observed coupling between executive and motor regions may reflect enhanced integration of cognitive control and motor planning mechanisms during cognitive–motor dual-task performance (Dørum et al., 2017; Heuninckx et al., 2008; O’Connell & Basak, 2018), consistent with intensified cognitive–motor interdependence in aging (Li & Lindenberger, 2002; Ren et al., 2013; Seidler et al., 2010). While we interpret this task-dependent increase in functional connectivity as potentially compensatory, given its association with preserved performance under higher demands (Geerligs et al., 2012), such patterns do not uniquely indicate compensation. Increased connectivity in older adults may alternatively reflect neural inefficiency or age-related dedifferentiation, whereby specialized neural systems become less distinct and more diffusely recruited (Argiris et al., 2024; Koen & Rugg, 2019; Martin et al., 2023).

By contrast, the motor-dominant network revealed a more distributed pattern of age-related reorganization under the same dual-task condition. Connectivity differences extended beyond frontal and parietal regions to include posterior cortical regions (e.g., precuneus, occipital cortex), subcortical structures (thalamus, putamen), and the cerebellum. Notably, older adults exhibited both strengthened (Old > Young) and weakened (Old < Young) connectivity. Stronger connectivity emerged in frontoparietal control regions, whereas reduced connectivity was observed in posterior and cerebellar–parietal pathways. These findings suggest that the motor-dominant aspect of dual tasking in aging involves a broader reorganization of functional networks, characterized by strengthened top–down control processes alongside reduced bottom–up sensorimotor integration.

To further clarify the nature of the network configurations examined in this study, we conducted an additional contrast isolating dual-task-specific connectivity patterns (Dual Task – [Single Motor + Single Cognitive]; Supplementary Fig. S4). This analysis revealed a distinct pattern of functional connectivity that was selectively expressed during dual-task performance and largely absent during single-task conditions. Notably, this pattern differed from both the motor-dominant (Dual Go – Single Cognitive) and Single Motor network configurations. This finding supports our conceptualization of motor-dominant, as well as cognitive-dominant networks as *context-dependent configurations* rather than isolable domain-specific modules. The motor-dominant network (Dual Go – Single Cognitive) captures motor processing as expressed within the dual-task context and, therefore, differs from the single-task motor baseline (SM contrast) and is not reducible to a simple additive combination of single-task components.

Such network-level changes closely parallel the behavioral finding of disproportionate motor dual-task costs and increased performance instability in older adults. In light of the reduced motor automaticity suggested by the behavioral results, the distributed connectivity changes observed in the motor-dominant network may reflect a greater reliance on higher-level control processes to sustain stable motor performance under dual-task demands. Together, these findings are consistent with prior work highlighting age-related shifts toward compensatory control and diminished sensorimotor efficiency (Bernard & Seidler, 2013; Seidler et al., 2010). They may also reflect increased processing demands or reduced efficiency of sensorimotor integration in aging (Goelman et al., 2023), highlighting the complexity of interpreting network-level reorganization during dual-task performance.

Importantly, our data highlight the cerebellum as a key locus of altered network dynamics in older adults under increasing dual-task demands. Once framed primarily as a motor structure, the cerebellum is now recognized as a key node in distributed networks supporting cognition across domains, particularly through its role in movement timing (Ivry et al., 2002; Schlerf et al., 2007), temporal prediction, coordination (Miall, 1998), and internal-model updating (Markov et al., 2021). This broader functional role is particularly relevant for aging-related neurological conditions such as Parkinson’s and Alzheimer’s, where posterior or vermal atrophy and altered cerebello-cortical coupling are often associated with early compensatory up-regulation (Liu et al., 2024; McElroy et al., 2024; B. Wang et al., 2025). In the single-task conditions, older adults showed mostly increased cerebellar connectivity (with some regional decreases in the motor task), suggesting that under moderate demands, the cerebellum may still provide compensatory support for motor–cognitive integration. This pattern aligns with meta-analytic evidence linking anterior cerebellar subdivisions to sequencing, temporal prediction, and internal-model processing, consistent with the modular organization of cerebellar contributions across task domains (Bernard & Seidler, 2013). However, under dual-task conditions, where integrative and coordination demands increase substantially, this pattern shifted toward reduced cerebello-cortical coupling. Older adults exhibited reduced coupling between the cerebellum and parietal regions as well as with anterior insular and cingulate cortices.

This shift may indicate age-related limitations in the cerebellum’s ability to support the temporal coordination and integration of motor responses under concurrent cognitive demands when task demands exceed compensatory capacity. In this context, the cerebellum’s normally supportive role may become insufficient when multiple task domains must be coordinated simultaneously. This interpretation aligns with lifespan accounts proposing a gradual shift in the cerebellum’s role from a primary driver of learning and coordination in childhood to a more supportive integrative role in adulthood, leaving older adults particularly vulnerable when task demands exceed available compensatory reserves (Beuriat et al., 2022). Structurally, recent diffusion tensor imaging evidence shows accelerated age-related decline in the cerebello-thalamo-cortical tract, with reduced white matter integrity predicting poorer executive function from late middle age onward (Kraft et al., 2025). Together with evidence for declining long-range functional hubs and compensatory increases in cerebellar connectivity with age (Tomasi & Volkow, 2012), our findings suggest that the cerebellum may represent both a site of early adaptive reorganization and a critical bottleneck for maintaining dual-task performance in later life. Our data, therefore, extend compensatory-recruitment models by highlighting task-dependent vulnerability: supportive contributions are evident under single-task demands, but integrative dynamics degrade under dual-task load. Together, these findings suggest that age-related changes in dual-task performance are not simply the result of generalized decline, but also reflect a reorganization of large-scale brain networks in which cognitive control systems remain relatively resilient while motor-related networks become increasingly dependent on higher-level control under increasing coordination demands.

### 4.4 Latent brain–behavior dimensions reveal age-specific patterns

To examine how reorganization of functional connectivity relates to behavior across multiple measures in an integrated manner, we applied a multivariate approach (RGCCA) (Rohart et al., 2017; Tenenhaus et al., 2017; Welham et al., 2023). This method identifies latent components that maximize shared variance between blocks of variables. The first RGCCA component (Fig. 7) captured a common gradient along which age groups showed a tendency toward separation in a domain- and task-dependent manner.

In the cognitive-dominant network, age groups showed clear separation along Component 1 during the single-task condition, although this separation was less pronounced than in the motor-dominant network, indicating smaller age-related differences in the cognitive domain. Under dual-task conditions, older adults clustered more tightly at the lower end of the gradient, resulting in largely overlapping score distributions across age groups. Notably, this overlap was more pronounced than that observed in the motor-dominant network under the same dual-task conditions. This pattern is consistent with our behavioral findings, in which older adults exhibited slower baseline RT in the single cognitive task but showed no significant age-group differences under dual-task conditions. Because dual-task effects are expressed relative to baseline performance, slower baseline processing speed in older adults may attenuate apparent group differences under increased task demands. At the same time, the convergence observed at the multivariate level—encompassing both behavioral and functional connectivity patterns—suggests a task-dependent alignment of brain–behavior organization across age groups.

One possible interpretation is that increasing task demands elicit a qualitative reorganization of functional brain networks, shifting older adults toward a more task-optimized configuration. Prior work has shown that task engagement is associated with substantial reconfiguration of network architecture, particularly within frontoparietal systems supporting cognitive control (Braun et al., 2015), and that such dynamic reconfiguration is behaviorally relevant and supports task performance (Bassett et al., 2011). In this context, the pronounced age-related differences observed during single-task performance may reflect variability in initial network organization. However, these differences were reduced under dual-task conditions, where both behavioral and connectivity patterns showed greater overlap between age groups. This pattern is consistent with evidence that, following initial task-induced reorganization, further increases in task demands primarily modulate or stabilize existing network configurations, rather than inducing additional large-scale reconfiguration (Hahn et al., 2020).

In the motor-dominant network, older adults displayed greater dispersion in component scores during single-task performance, suggesting heterogeneous reorganization strategies even under relatively low motor demands. Behaviorally, the absence of significant age differences in single-task motor RT is likely related to the relatively slow cycling cadence imposed by the MR environment (~4.3 s), which reduced baseline motor demands compared with natural locomotion (~1.1 s cadence). Under dual-task conditions, older adults clustered more tightly at the lower end of the gradient; however, group separation remained more pronounced than in the cognitive-dominant network, consistent with the larger age-related differences observed in motor dual-task RT and variability. These findings indicate that motor systems may be more sensitive to age-related constraints, whereas cognitive systems may retain greater capacity for adaptive reorganization, leading to more similar brain–behavior alignment across age groups under increased task demands (Grande et al., 2023; Song et al., 2014; Ward & Frackowiak, 2003).

To further characterize how functional connectivity and behavior jointly align along the latent brain–behavior dimension captured by Component 1, we examined block-specific component scores and their pairwise correlations. In the motor-dominant network, younger adults showed stronger correlations between single- and dual-task connectivity, whereas these associations were weaker in older adults. This pattern suggests that functional connectivity in older adults undergoes greater reconfiguration from single- to dual-task conditions, transitioning from more variable initial network organization toward a more stabilized or modulated configuration under increased task demands. Furthermore, correlations between functional connectivity and behavioral performance tended to be higher in older adults than in younger adults, particularly under dual-task conditions. This pattern suggests that behavioral performance in older adults may be more tightly coupled to the configuration of large-scale functional networks. Notably, correlations with single-task connectivity were comparable in strength to those observed for dual-task connectivity, indicating that even under simpler conditions, functional connectivity captures network dynamics that are relevant for dual-task performance. Such stronger brain–behavior coupling in older adults may also help explain why variability in motor performance emerged as the dominant behavioral contributor to the latent brain–behavior dimension.

Post hoc examination of the behavioural loadings revealed that measures of response time variability—particularly motor dual-task variability—contributed most strongly to this latent dimension. Motor dual-task cost in mean RT (Dual.task.cost_RT_motor) also showed substantial contributions, together with several other variability-related measures. The corresponding connectivity loadings further demonstrated that this behavioral dimension was most strongly associated with connections linking motor planning and cognitive control regions, particularly the anterior supramarginal gyrus (aSMG), supplementary motor area (SMA), and precentral gyrus. The aSMG has been implicated in sensorimotor and conceptual representations of motor actions. Neuroimaging studies show that the anterior supramarginal gyrus is recruited during the processing of action verbs and affordances, suggesting that this region may activate motor representations associated with action execution (Di Cesare et al., 2017). Similarly, mental simulation of actions and action-related language recruit overlapping fronto–parietal networks including inferior parietal regions, indicating that these areas contribute to mental representations of actions (Péran et al., 2010). These findings are consistent with coordinated interactions between parietal regions involved in sensorimotor and action representations (e.g., aSMG) and medial frontal motor areas involved in motor planning, sequencing (e.g., SMA), and processes that may facilitate the coordination of concurrent cognitive and motor task demands (Bonini et al., 2014; Cona & Semenza, 2017; Courson et al., 2017).

Taken together, these multivariate findings extend the univariate connectivity results by showing that age-related differences are expressed not only in mean connectivity strength but also in the structure and stability of brain–behavior coupling. Consistent with theories of intensified cross-domain interdependence in aging (Huxhold et al., 2009; Li & Lindenberger, 2002), the greater variability and dispersion observed in motor-dominant networks may reflect an increased reliance of motor stability on attentional and executive control resources. Rather than indicating isolated motor dysfunction, these latent dimensions suggest a pattern of asymmetric reorganization across systems: motor-related networks appear more susceptible to performance fluctuations under increasing coordination demands, whereas cognitive networks show relatively stable integration at the level of dual-task performance.

This interpretation aligns with recent work on functional connectome gradients showing that aging affects large-scale network organization in a non-linear, system-specific manner (Riccardi et al., 2025; Tomasi & Volkow, 2012; X. Wang et al., 2024). Within this framework, sensory–motor networks often exhibit greater dedifferentiation or variability with age, whereas higher-order cognitive networks may retain relatively stable functional organization under moderate task demands. The present findings, showing greater dispersion and vulnerability in motor-dominant networks alongside relatively preserved organization in cognitive-dominant networks, are broadly consistent with these observations of selective resilience and dedifferentiation across functional systems. These findings, therefore, support the view that aging does not uniformly degrade functional networks but instead reshapes their hierarchical organization, with motor systems showing greater sensitivity to fluctuations in control demands than cognitive systems under the conditions examined here. Overall, the present results suggest that cognitive–motor dual tasking in aging reflects a reconfiguration of large-scale functional networks, with preserved executive systems increasingly recruited to support declining motor stability under increasing coordination demands.

### 4.5 Limitations

Several limitations should be considered when interpreting the present findings. The sample consisted of relatively healthy, well-educated, right-handed younger and older adults, which may limit generalizability to more diverse or clinical populations. In addition, commonly used clinical screening measures such as the Montreal Cognitive Assessment (MoCA) and objective overground walking-speed assessments were not collected, although executive and attentional functioning were evaluated using the TAP-M battery and functional mobility was partially characterized using SF-12 items. Second, the experimental paradigm prioritized control over ecological validity. The motor task involved supine pedaling rather than overground walking, which reduces several key motor and sensory demands, including requirements for balance and postural control, integration of vestibular and proprioceptive inputs, and spatial navigation. Although the externally paced pedaling ensured temporal alignment with the cognitive task and minimized motion artifacts, it likely reduced motor automaticity compared with natural walking. Consequently, the observed motor and cognitive–motor effects should be interpreted cautiously when generalizing to real-world walking. The cognitive task of the paradigm relied on a relatively easy Go/NoGo task. Although age-related slowing was evident in the sample, particularly in more demanding attentional measures of the TAP-M battery administered outside the scanner, the in-scanner task may have imposed relatively modest cognitive demands and, therefore, been less sensitive to subtle age-related deficits under dual-task conditions. Third, increased functional connectivity in older adults was interpreted as reflecting compensatory network recruitment based on task dependence and preserved behavioral performance. However, such increases may also reflect neural inefficiency or age-related dedifferentiation. Distinguishing adaptive reorganization from reduced network specificity will require multimodal and longitudinal approaches, to determine whether the connectivity patterns identified here predict future cognitive or motor decline. Despite these limitations, the present design provides a controlled framework for characterizing how cognitive–motor task demands shape age-related differences in brain network organization, offering a foundation for future studies using more naturalistic behavioral paradigms.

### 4.6 Future directions

Future studies adopting multimodal approaches will be critical for clarifying the functional significance of increased connectivity in aging. In addition, integrating more demanding cognitive paradigms (e.g., higher-load divided attention or task-switching tasks) with naturalistic gait assessments may provide a more comprehensive characterization of cognitive–motor interactions in aging. For example, studies combining overground dual-task walking with structural MRI have demonstrated widespread age-related atrophy and white matter degradation, particularly in sensorimotor regions, and have linked these structural changes to dual-task walking performance (Hupfeld et al., 2022). While such work provides important insight into structural constraints on dual-task performance, the absence of task-related fMRI highlights the need for integrated longitudinal designs that combine naturalistic behavior with both structural and functional neuroimaging (Bunzeck et al., 2024). Such approaches would allow stronger inferences regarding whether observed increases in functional connectivity reflect adaptive compensation, reduced neural efficiency, or age-related dedifferentiation.

## 5 Conclusion

This study demonstrates that aging differentially impacts large-scale brain networks supporting cognitive–motor dual tasking. While cognitive networks remained relatively resilient, maintaining dual-task performance through focused frontal upregulation, motor-dominant networks exhibited broader and more variable reconfiguration. This reconfiguration, characterized by strengthened top–down frontoparietal control alongside reduced efficiency of bottom–up sensorimotor and cerebellar pathways, suggests that motor performance in older adults becomes increasingly dependent on central control resources.

These findings refine current models of cognitive–motor aging by showing that age-related decline is not uniform but reflects a reweighting of neural dependencies across functional systems. Preserved cognitive performance in older adults appears to occur alongside—and possibly as a consequence of—this neural shift, highlighting an adaptive yet constrained process. In this context, motor variability, closely linked to frontoparietal control networks, emerged as a key behavioral signature of this vulnerability. Measures of motor stability and cognitive–motor interdependence may, therefore, provide sensitive markers for identifying individuals at risk of mobility decline, with potential implications for both basic aging research and the early detection of neurodegenerative conditions. Future longitudinal studies will be essential to determine whether these network-level patterns predict subsequent changes in mobility and cognitive–motor function, and whether they can inform the development of targeted interventions for healthy aging. More broadly, understanding how aging reshapes the balance between cognitive control and sensorimotor efficiency may be critical for explaining why cognitive–motor abilities remain stable in some individuals while declining in others.

## Supplementary Material

Supplementary Material

## Data Availability

The broader research project was preregistered on the Open Science Framework (OSF): https://osf.io/pjt2x/overview. Preprocessed task-based fMRI data are publicly available via OSF: https://osf.io/pjt2x/resources. Analysis code is available on GitHub: https://github.com/ydeng2021/dual-task-fmri-aging-RGCCA-analysis and is distributed under the MIT License. While the preregistration specifies the general study design and hypotheses, the analyses reported here represent a subset of the preregistered framework and include additional methodological extensions.
